# Kinase condensates enrich ATP and trigger autophosphorylation

**DOI:** 10.1016/j.celrep.2026.117459

**Published:** 2026-06-01

**Authors:** Nicholas E. Lea, Lindsay B. Case

**Affiliations:** 1 Department of Biology, Massachusetts Institute of Technology, Cambridge, MA, USA; 2 Lead contact

## Abstract

Kinase-mediated signal transduction regulates most cellular processes, and concentration-dependent autophosphorylation is a common mechanism to promote kinase signaling. Many kinases undergo phase separation to form condensates. Despite the central role of autophosphorylation in regulating kinase activity, how condensates impact kinase autophosphorylation has not been systematically studied. Using biochemical reconstitution and cellular studies, we find that phase separation can concentrate kinases to effectively trigger the *trans*-autophosphorylation of the tyrosine kinases FAK and Abl, as well as the serine/threonine kinase Mst2. Moreover, kinase condensates can create a chemical environment that enriches ATP, and positively charged intrinsically disordered regions are one feature that enrich ATP into condensates. Thus, kinase phase separation is a general mechanism to activate kinase signaling pathways by locally concentrating both kinases and ATP to trigger autophosphorylation.

## In brief

Lea et al. find that some kinases form highly concentrated condensates that enrich ATP through electrostatic potential and significantly increase rates of *trans*autophosphorylation. Condensates that enrich ATP and concentrate kinases may be a broad mechanism to initiate and sustain kinase signaling in both normal cells and in cancer.

## Graphical Abstract

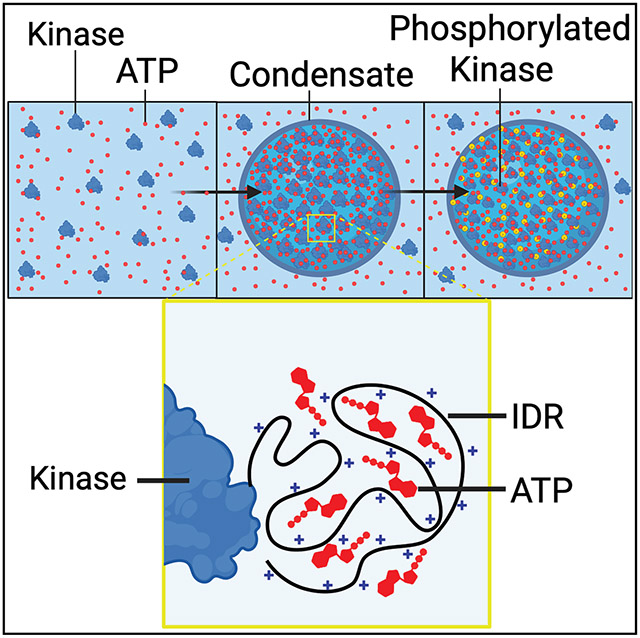

## INTRODUCTION

**Figure 1.**
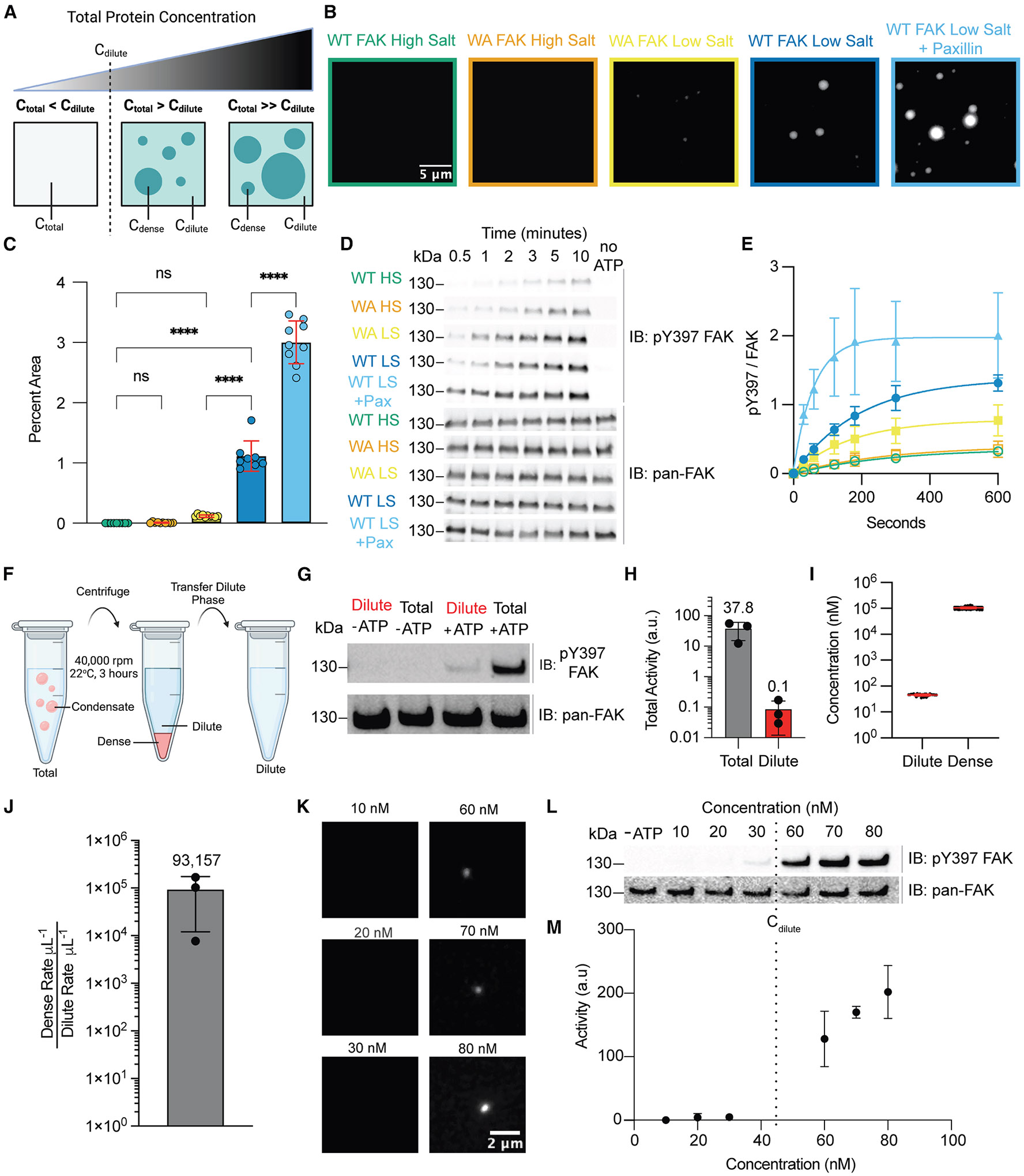
FAK condensates trigger autophosphorylation in vitro (A) Schematic of concentration-dependent phase separation for a single protein system. (B) Images of 1 μM mEGFP-FAK with different buffers or mutations. Buffer: 25 mM HEPES pH 7.5, 50 (low salt) or 300 (high salt) mM NaCl, 1% glycerol, 1 mM DTT. Paxillin is 100 nM. Scale bars, 5 μm. (C) Quantification of percent area occupied by condensates for conditions in (B). n > 7 images per condition. Significance was tested with Brown-Forsythe and Welch ANOVA with Dunnett’s T3 multiple comparison tests (*p < 0.0332, **p < 0.0021, ***p < 0.0002, ****p < 0.0001). (D) Western blots of FAK autophosphorylation for each condition in (B). (E) Quantification of data in (C). n = 3 replicates. (F) Diagram of separating condensates by centrifugation. (G) Western blot of total and dilute phase initial autophosphorylation rates at 30 s. (H) Quantification of data in (G). n = 3 replicates. (I) Measurements of mEGFP-FAK dilute phase concentration and dense phase concentration using fluorescence microscopy. n = 34 images for C_dilute_ and n = 502 droplets for C_dense_. (J) Volume-normalized fold rate enhancement of dense phase. (K) Images of mEGFP-FAK. Buffer matches low salt buffer in (B). Scale bars, 2 μm. (L) Western blot of FAK autophosphorylation. (M) Quantification of the data in (L) (n = 3 replicates). Dotted line represents C_dilute_ value measured in (I). For all graphs, error bars are standard deviation and numbers above error bars are means. Also see Figures S1, 2, and 3.

Kinase activity plays a central role in signal transduction and regulates most cellular processes. High-fidelity signal transduction requires kinase activity to be tightly regulated, ensuring that substrate phosphorylation occurs at the correct time and place. Growing evidence suggests eukaryotic cells can regulate signal transduction by recruiting kinases to biomolecular condensates.^1-3^ Condensates are non-membrane-bound cellular compartments that concentrate specific biomolecules and regulate diverse cellular processes.^4-6^ Many condensates form through the phase separation of their constituent macromolecules, a thermodynamically spontaneous, favorable process often driven by weak, multivalent protein interactions mediated by folded domains and intrinsically disordered regions (IDRs).^7^ Phase separation depends on molecular concentrations as well as solution conditions such as temperature, pH, salt concentration, and molecular crowding. Phase separation results in two distinct phases, a “dense phase” highly concentrated in select molecules and a “dilute phase” that is much less concentrated.^5^ As total protein concentration increases, the dense phase concentration and dilute phase concentration remain constant, and the dense phase volume increases (Figure 1A). In addition to concentrating and excluding specific macromolecules, condensates have emergent physical and chemical properties that can be important for their function.^7-10^

Many kinases directly undergo phase separation to drive condensate formation.^6^ These kinase condensates often promote signaling and regulate various cellular processes. Wnk kinase phase separation regulates cell-volume homeostasis,^11^ ATG1 phase separation promotes autophagasome assembly,^12^ MST (Mammalian STE20-like protein kinase) and LATS (Large tumor supporessor homolog) phase separation regulates proliferation through the Hippo signaling pathway,^13^ and the RI*α* subunit of PKA undergoes phase separation to regulate cell survival.^14^ Phosphorylated receptor tyrosine kinases, including FGFR and EGFR, can phase separate through multivalent interactions with cytosolic adapter proteins to regulate downstream pathways controlling cell growth, survival, and proliferation.^15,16^ In cancer, chromosomal rearrangements can result in oncogenic kinase fusion proteins that form aberrant cytoplasmic condensates that hyperactivate signaling.^14,17-19^ EML4-ALK fusion proteins and NTRK fusion proteins form cytoplasmic condensates that can activate tyrosine kinase activity, recruit downstream signaling proteins, and activate Ras-MAPK signaling in cancer.^17-19^ The DnaJB1-PKA_cat_ fusion protein forms condensates that disrupt RI*α* condensates causing defective cAMP localization and increased proliferation in liver cancer.^14^

Kinase condensates can impact signaling through a variety of mechanisms. Concentrating substrates and kinases together within synthetic condensates is sufficient to increase substrate phosphorylation and broaden kinase specificity.^20^ For Plk4 and PKA, the active conformation of the kinase is recruited to condensates.^14,21^ In the Hippo signaling pathway, positive and negative regulators of the Hippo kinase cascade can both undergo phase separation.^13^ Positive regulators of Hippo signaling form condensates that locally concentrate kinases and increase their signaling, whereas negative regulators of Hippo signaling form condensates that inhibit signaling. Condensates can regulate kinase signaling through the recruitment or exclusion of proteins and small molecules. In the Hippo pathway, SLMAP condensates that recruit MST and the STRIPAK phosphatase complex inhibit signaling.^13^ During T cell activation, LAT condensates exclude negatively charged phosphatases to sustain signaling.^10^ RI*α* condensates recruit cAMP through high-affinity binding to the structured cAMP-binding domains, leading to high PKA activity inside condensates.^14^ The solid-like property of Merlin condensates are required to activate Hippo signaling, demonstrating that the emergent properties of kinase condensates also influence signaling.^8^ Kinase condensates can also modulate the activity of downstream signaling molecules. FGFR2 forms condensates containing the phosphatase SHP2 and the phospholipase PLC*y*1.^15^ Inside the condensate, kinase and phospholipase activities are increased while phosphatase activity is decreased. EGFR condensates can increase Ras activation by the guanine nucleotide exchange factor SOS.^16^ Although the signaling activity of kinase condensates can be positively and negatively regulated through many mechanisms, how kinase condensates specifically impact kinase autophosphorylation has not been systematically studied.

Autophosphorylation is a common mechanism to initiate signaling of eukaryotic kinases, and kinases have diverse structural mechanisms for achieving autophosphorylation.^22,23^ Autophosphorylation that occurs intramolecularly does not depend on kinase concentration. For many kinases, autophosphorylation can occur through bimolecular mechanisms that are concentration dependent, such as *trans*-autophosphorylation. Since phase separation rapidly increases protein concentration, we hypothesized that kinase condensates could promote concentration-dependent autophosphorylation. Using biochemical reconstitution and cellular studies, we find that phase separation can concentrate kinases to effectively trigger the autophosphorylation of cytoplasmic tyrosine and serine/threonine kinases that undergo concentration dependent *trans*-autophosphorylation. Moreover, kinase condensates can create a chemical environment that enriches adenosine triphosphate (ATP). For some proteins, IDRs with a net positive charge are both necessary and sufficient to drive high levels of ATP enrichment into condensates. Our data suggest that kinase phase separation is a general mechanism to activate kinase signaling pathways by locally concentrating both kinases and ATP, robustly triggering autophosphorylation.

## RESULTS

### FAK condensates trigger autophosphorylation *in vitro*

Focal adhesion kinase (FAK) is a cytoplasmic tyrosine kinase that regulates integrin-dependent survival and migration.^24^ FAK is typically autophosphorylated at focal adhesions, supramolecular assemblies that form at the plasma membrane when integrin receptors bind the extracellular matrix (ECM). FAK autophosphorylation creates a binding site for Src kinase, leading to phosphorylation of additional substrates and downstream signaling.^25^ We previously found that purified full-length FAK undergoes spontaneous phase separation to form micron-sized liquid-like condensates at nanomolar concentrations.^26^ FAK phase separation is driven by weak, multivalent, intermolecular interactions^26^ including dimerization of the FERM domain (29 μM affinity^27^), electrostatic interaction between the FERM domain and C-terminal FAT domain (0.6 μM affinity^27^), and dimerization mediated by a 200 amino acid IDR (0.85 μM affinity^28^). FAK phase separation is enhanced by the adapter protein paxillin, due to multivalent interactions between paxillin LD motifs and the FAT domain of FAK.^26^ FAK phase separation contributes to initial focal adhesion formation in cells spreading on ECM.^26^

FAK-dependent signaling requires FAK autophosphorylation on tyrosine 397 (Y397), which predominantly occurs *in trans*.^29^ Autophosphorylation of full-length FAK is concentration-dependent *in vitro*,^27^ and artificially dimerizing FAK with a small molecule induces autophosphorylation in cells.^29^ In this study, we used recombinant full-length FAK as a simple model system to study concentration-dependent autophosphorylation in the context of phase separation (Figure S1A). We found that both phosphorylated and dephosphorylated FAK undergo phase separation, although phosphorylated FAK has a slightly increased dense phase volume at similar concentrations (Figures S1B-S1D). Thus, tyrosine phosphorylation is not required for FAK phase separation and autophosphorylation does not dramatically change FAK phase separation propensity.

To test if phase separation changes the rate of FAK autophosphorylation, we used perturbations that reduce or enhance FAK phase separation. As previously reported,^26^ NaCl concentrations of 300 mM completely inhibit phase separation by preventing electrostatic interactions between the FERM and FAT domains, a mutation of the FERM domain dimerization interface (W266A) decreases phase separation by reducing FAK dimerization, and the addition of 100 nM paxillin increases phase separation by introducing additional multivalent protein interactions (Figures 1B and 1C). From microscopy images, we quantified the percentage of pixels contained within condensates. Condensate percent area depends on both condensate size and number and is a proxy for the total dense phase volume. If we maintain FAK at 1 μM concentration, the condensate percent area significantly varies across the conditions tested (Figure 1C). We measured FAK autophosphorylation kinetics using a phosphospecific antibody for pY397 and found that conditions with increased condensate percent area exhibit faster rates of autophosphorylation (Figures 1D and 1E). In high salt buffer, neither WT nor W266A FAK undergoes phase separation, and we measure identical, slow rates of autophosphorylation with both proteins. This suggests WT FAK does not form stable dimers under these conditions, consistent with the previously measured 29 μM dimerization affinity. In low salt buffer, both WT and W266A FAK undergo phase separation, but WT FAK has significantly increased condensate percent area compared to W266A FAK. WT FAK also undergoes faster autophosphorylation in these conditions. These data demonstrate that FERM domain dimerization is not specifically required for FAK autophosphorylation in condensates.

Since increasing the dense phase volume resulted in faster rates, we hypothesized that FAK localized within the dense phase undergoes autophosphorylation faster than FAK in the dilute phase. To test this hypothesis, we used a previously developed sedimentation assay^30^ to directly measure the dilute phase rate and indirectly assess the dense phase rate (Figure 1F). We observed that the initial rate of autophosphorylation is substantially slower in the dilute phase compared to the total solution and estimate that the dense phase accounts for >99% of the total solution activity (Figures 1G and 1H). Using quantitative microscopy, we found that the mEGFP-FAK concentration is 45 nM in the dilute phase and 106 μM in the dense phase (Figure 1I). Under these experimental conditions, >95% of FAK molecules are localized in condensates. Although FAK condensates account for most of the solution activity, they occupy <1% of the solution volume in these assays. For a given volume, we estimate that the autophosphorylation rate is ~90,000 times faster within condensates compared to the dilute phase (Figure 1J; see STAR Methods for details).

Phase separation occurs at concentrations above the saturation concentration (C_sat_), which is also the concentration of protein in the dilute phase at equilibrium (C_dilute_, Figure 1A).^31^ The mEGFP-FAK C_dilute_ is 45 nM, which we validated independently with quantitative microscopy (Figure 1I), quantitative western blots (Figures S2A-S1C), and titration (Figures 1K and S2D). When we titrated mEGFP-FAK across its C_dilute_, we detect no condensates at 30 nM but numerous condensates at 60 nM (Figure S2D). Additionally, we performed mass photometry on mEGFP-FAK and found there is no change in FAK oligomerization in the dilute phase across these concentrations (Figure S2E). Using multiplex fluorescent western blots run with on-blot pFAK standards to accurately measure FAK autophosphorylation, we observed a 10- to 25-fold increase in autophosphorylation activity between concentrations just below and just above C_dilute_ (30 nM vs. 60 nM) (Figures 1L-1M; Figures S3A-S3C). This increase in autophosphorylation is more than expected from doubling the total FAK concentration, consistent with FAK condensates, which only form above 45 nM total FAK concentration, having drastically increased autophosphorylation rates due to the high dense phase concentration (~100,000 nM). We conclude that the rate of autophosphorylation is significantly higher in the dense phase. Together, these biochemical experiments demonstrate that phase separation is sufficient to trigger robust FAK *trans*-autophosphorylation on Y397 by locally increasing kinase concentration by three orders of magnitude.

### FAK condensates trigger autophosphorylation in cells

**Figure 2.**
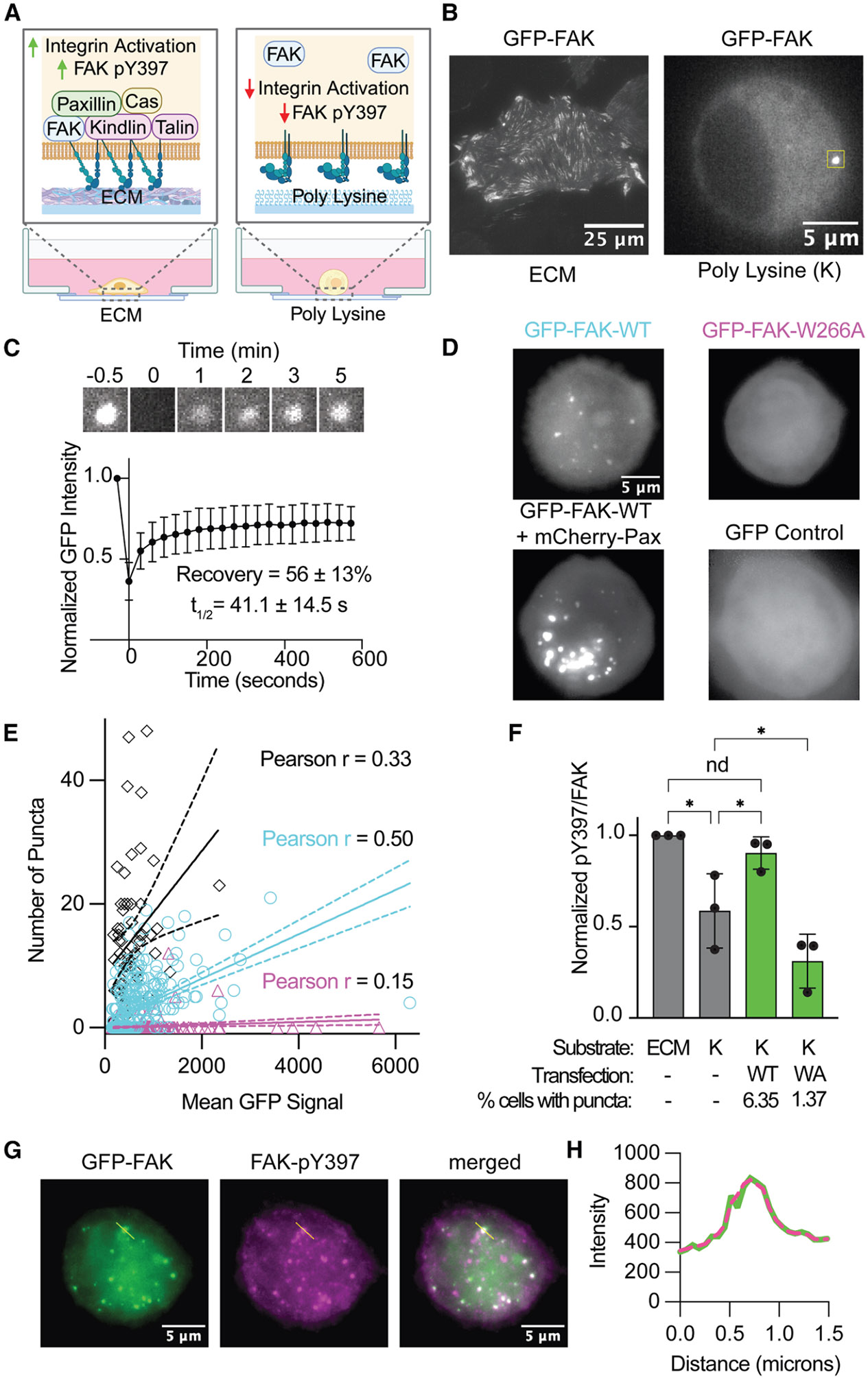
Cytoplasmic FAK condensates are sufficient to increase FAK autophosphorylation independent of integrin-based adhesion (A) Diagram of MEFs plated on extracellular matrix (ECM) or poly-D-Lysine. (B) Representative images of mEGFP-FAK in MEFs. (C) Fluorescent Recovery After Photobleaching (FRAP) of the GFP-FAK puncta in (B) and FRAP analysis for n = 5 replicates. (D) Representative maximum intensity projections of MEFs expressing GFP-tagged proteins. (E) Quantification of number of puncta in cells vs. total GFP signal (n > 60 cells per condition from two experimental replicates). Dotted lines represent 95% confidence intervals of linear regressions. GFP signal allows accurate relative comparisons of GFP-tagged protein concentration between cells but not absolute quantification of protein concentration. (F) pY397/FAK ratios determined by western blot analysis of MEF lysates. K denotes cells plated on poly-D-Lysine for 30 min. ECM denotes cells grown on culture treated dishes for 24 h. Green bars indicate analysis was performed on mEGFP-FAK and gray bars on endogenous FAK. Significance was tested with one-way ANOVA with correction for multiple comparisons using the two-stage linear step-up procedure of Benjamini, Krieger, and Yekutieli (*p < 0.05). (G) Immunostain images of MEFs expressing mEGFP-FAK and plated on poly-D-Lysine. n = 3 replicates. (H) Linescan intensity of yellow line in (G). All data show mean ± standard deviation. Scale bars, 5 or 25 μm (indicated on images). Also see Figures S4, 5, and S6-S8.

We next sought to test if FAK phase separation is sufficient to trigger FAK autophosphorylation in cells. The cellular concentration of FAK is estimated to be 5–10 nM in fibroblasts.^27^ Thus, endogenous FAK is maintained at concentrations below its C_sat_, and FAK phase separation is likely regulated by its local concentration at focal adhesions through protein and lipid interactions.^26,32^ However, we hypothesized that overexpression of FAK to concentrations above its C_sat_ would promote spontaneous FAK phase separation in the cytoplasm. To test this, we transiently overexpressed mEGFP-FAK in mouse embryonic fibroblasts (MEFs). To prevent integrin-dependent FAK autophosphorylation, we plated cells on poly-D-Lysine coated plates for 30 min to allow cells to attach to the surface without integrin adhesion (Figure 2A). We found that cells expressing high levels of mEGFP-FAK contained cytoplasmic mEGFP-FAK puncta (Figure 2B). Although FAK can associate with and signal from endosomes,^33^ these cytoplasmic FAK puncta did not co-localize with recycling (Rab11 positive) or early (EEA1 positive) endosomes (Figures S4A and S4B). Cytoplasmic mEGFP puncta exhibit dynamic fluorescence recovery after photobleaching (FRAP) inconsistent with solid aggregation (t_1/2_ = 41.1 ± 14.5 s; 56 ± 13% recovery) (Figure 2C). Cytoplasmic puncta are slightly more dynamic than condensates *in vitro* (t_1/2_ = 64.5 ± 10.8 s; 35 ± 7% recovery) (Figures S5A and S5B), while less dynamic than focal adhesions at the plasma membrane (t_1/2_ = 9.8 ± 7.0 s; 57 ± 11% recovery) (Figure S5C). We conclude that FAK overexpression is sufficient to form dynamic cytoplasmic puncta distinct from endosomes.

To further investigate whether cytoplasmic FAK puncta are condensates formed through phase separation, we tested the same perturbations previously characterized *in vitro* (Figure 1B). Similar to *in vitro* experiments, mEGFP-FAK-W266A forms significantly fewer puncta than mEGFP-FAK-WT. Additionally, co-expressing mEGFP-FAK-WT or mEGFP-FAK-W266A with mCherry-Paxillin significantly increases the number of mEGFP puncta (Figures 2D-2E; Figures S6A-S6D). The addition of mCherry-Paxillin also substantially increases the percent of transfected cells that contained puncta for both wild type and W266A FAK (Figures S6E and S6F). The number of puncta in a cell correlates positively with mEGFP-FAK expression levels, consistent with concentration-dependent phase separation (Figures 2E; S6D). Occasionally, we observed large, non-spherical aggregates in cells with the highest mEGFP-FAK-WT expression levels (Figure S6E). We conclude that overexpressing FAK above the C_sat_ is sufficient to induce spontaneous condensate formation in the cytoplasm through the same interactions that drive FAK phase separation *in vitro*.

We next tested if cytoplasmic FAK condensates are sufficient to promote autophosphorylation in cells. As previously reported, cells plated on poly-D-Lysine for 30 min have reduced pY397/total FAK ratios compared to cells plated on tissue culture plates for 24 h, enough time to secrete ECM and form integrin-based adhesions (Figure 2F).^34^ However, overexpressing mEGFP-FAK-WT in cells at levels that induce cytoplasmic condensates in 6.4% of cells is sufficient to rescue autophosphorylation on poly-D-Lysine (Figure 2F), consistent with cytoplasmic FAK condensates increasing autophosphorylation in cells. In contrast, overexpressing mEGFP-FAK-W266A, which rarely forms condensates, did not rescue autophosphorylation (Figure 2F). Moreover, expressing mEGFP-FAK-WT at lower levels (~1.5-fold above endogenous) that do not induce cytoplasmic condensates does not rescue autophosphorylation, indicating that FAK overexpression does not increase cellular pY397/FAK ratios in the absence of condensates (Figure S7). Although cytoplasmic FAK condensates promote FAK autophosphorylation, we did not observe statistically significant increases in downstream paxillin or p130Cas phosphorylation 30 min after plating on poly-D-Lysine (Figures S8A and S8B). In the mEGFP-FAK-WT-transfected cells, p130Cas phosphorylation was higher than untransfected cells; however, it did not reach the statistical significance threshold (*p* = 0.27). Since fibroblasts secrete ECM, untransfected cells had fully recovered integrin-dependent Y410 p130Cas phosphorylation by 60 min after plating on poly-D-Lysine (Figures S8C and S8D). Thus, analysis of condensate-specific signaling at later time points is not possible in this assay. Finally, we performed immunostaining and found that cytoplasmic FAK condensates contain pY397 FAK (Figures 2G and 2H). We conclude that cytoplasmic FAK condensates are sufficient to trigger FAK autophosphorylation even in the absence of upstream integrin activation.

### Abl and Mst2:Sav1 condensates trigger autophosphorylation *in vitro*

**Figure 3.**
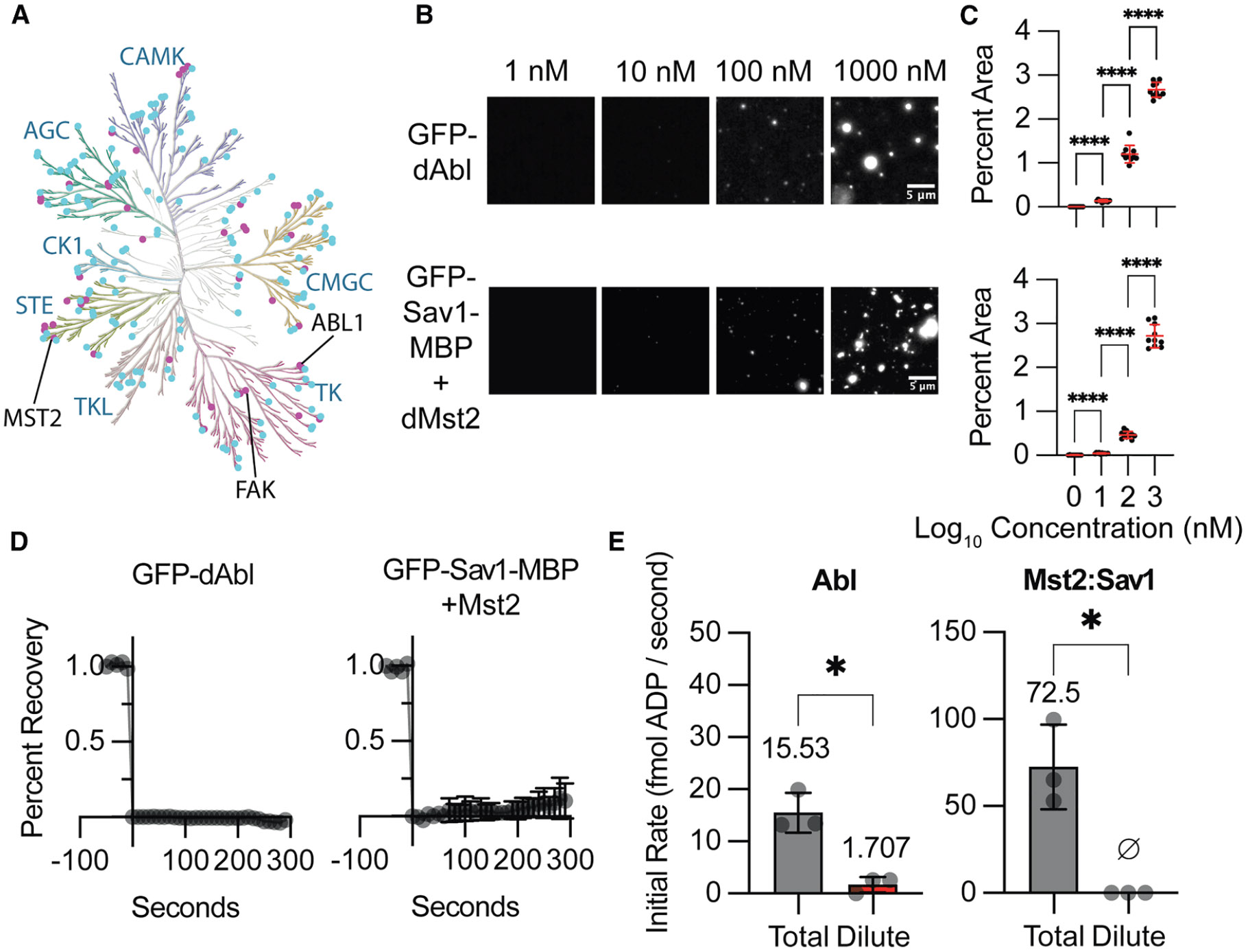
Abl and Mst2:Sav1 condensates trigger autophosphorylation in vitro (A) Homology dendrogram of the human kinome. Kinases predicted to phase separate are denoted by colored dots (at least one predictor = cyan; all three predictors = magenta). (B) Images of mEGFP-dAbl and mEGFP-Sav1-MBP + dMst2. mEGFP-Sav1-MBP and dMst2 are kept at equimolar concentrations. Abl buffer is 25 mM HEPES pH 7.5, 100 mM NaCl, 5% PEG8000, 1% glycerol (v/v), 1 mM DTT. Sav1:Mst2 buffer is 25 mM HEPES pH 7.5, 100 mM NaCl, 10% PEG8000, 1 mM DTT. Scale bars, 5 μm. (C) Quantification of percent area occupied by condensates for conditions in (A). n = 10 images per condition. Significance was tested with Brown-Forsythe and Welch ANOVA with Dunnett’s T3 multiple comparison tests. (D) Fluorescence recovery after photo bleaching (FRAP) analysis of mEGFP-dAbl and mEGFP-Sav1-MBP + dMst2 condensates. mEGFP-dAbl is 1,000 nM. mEGFP-Sav1-MBP and dMst2 are 40 nM. Buffers same as (B). n = 5 replicates. (E) Initial rates of kinase phosphorylation assays. Buffers same as (B), except supplemented with 0.5 mM MgCl_2_ and initiated by addition of 1 μM ATP. n = 3 replicates. Number above error bars is the mean. ø symbol denotes activity was undetectable. Significance was tested with unpaired *t* test with Welch’s correction. For all graphs, error bars represent standard deviation. *p < 0.0332, **p < 0.0021, ***p < 0.0002, ****p < 0.0001. Also see Figures S9 and S10.

Based on our observations of FAK condensates, we hypothesized that phase separation would be sufficient to promote autophosphorylation of other kinases with concentration-dependent autophosphorylation mechanisms. Using three independent phase separation predictors developed with machine learning,^35-37^ we estimate that 13%–44% of human kinases have a strong likelihood of undergoing phase separation (Figure 3A; Data S1). 68 kinases (13% of human kinases) are predicted to phase separate by all three predictors, and 234 kinases (44% of human kinases) are predicted to phase separate by at least one predictor (Details in Data S1). To test if phase separation is a more general mechanism to trigger autophosphorylation, we characterized the phase separation and autophosphorylation of two additional kinases predicted to phase separate, Abl and Mst2, using biochemical approaches. Abl is a tyrosine kinase and proto-oncogene with a 460 amino acid IDR. Purified full-length Abl undergoes concentration-dependent autophosphorylation *in trans*, phosphorylating both Y393, in the activation loop, and Y226, in the autoinhibitory SH3-binding site, which both increase the activity of the kinase.^38^ Mst2kinase, also called Serine/threonine-prot is a serine/threonine kinase in the Hippo signaling pathway that phase separates when combined with the intrinsically disordered adapter protein Sav1.^13,39^ Purified Mst2 autophosphorylates *in trans* on T180 in the activation loop, which increases the activity of the kinase.^40^ Consistent with the phase separation prediction, we found that purified Abl undergoes concentration-dependent phase separation. Purified Sav1 also undergoes concentration-dependent phase separation to form condensates that enrich Mst2, as previously reported (Figures 3B and 3C; Figure S9).^39^ The exchange of protein between condensates and the dilute phase was much slower for Abl and Mst2-Sav1 condensates than for FAK condensates (Figure 3D). However, Hippo pathway activation has been shown to require solid-like condensates, suggesting less dynamic kinase condensates can activate signaling.^8^ We next used *in vitro* kinase assays to determine the phosphorylation activity in solutions containing condensates. We first confirmed the dephosphorylation and activity of purified Abl and Mst2 with phospho-specific antibodies to Y226 (Abl) and T180 (Mst2) (Figures S10A and S10B). We then used an ADPGlo luciferase assay for high-sensitivity and robust quantitation of phosphorylation rates in the total solution and dilute phase. Similar to FAK, the total solution of Abl and Mst2 showed fast initial rates of phosphorylation, while almost no phosphorylation occurred after removing condensates from the dilute phase (Figures 3E; S10C). We used phospho-proteomics analysis to determine the specific residues that were modified in these experiments (Figures S10D-S10G). While the only phosphotyrosine detected on FAK was the canonical autophosphorylation site,^34^ Abl and Mst2 phosphorylated additional residues beyond their known autophosphorylation sites.^38,40^ This observation suggests that condensates expand the specificity of autophosphorylation for some kinases, consistent with previous studies demonstrating that synthetic kinase condensates can phosphorylate unexpected peptides.^20^ We conclude that phase separation is a general mechanism that can trigger *trans*-autophosphorylation of both tyrosine and serine/threonine kinases.

### Kinase condensates enrich ATP independent of active site binding

**Figure 4.**
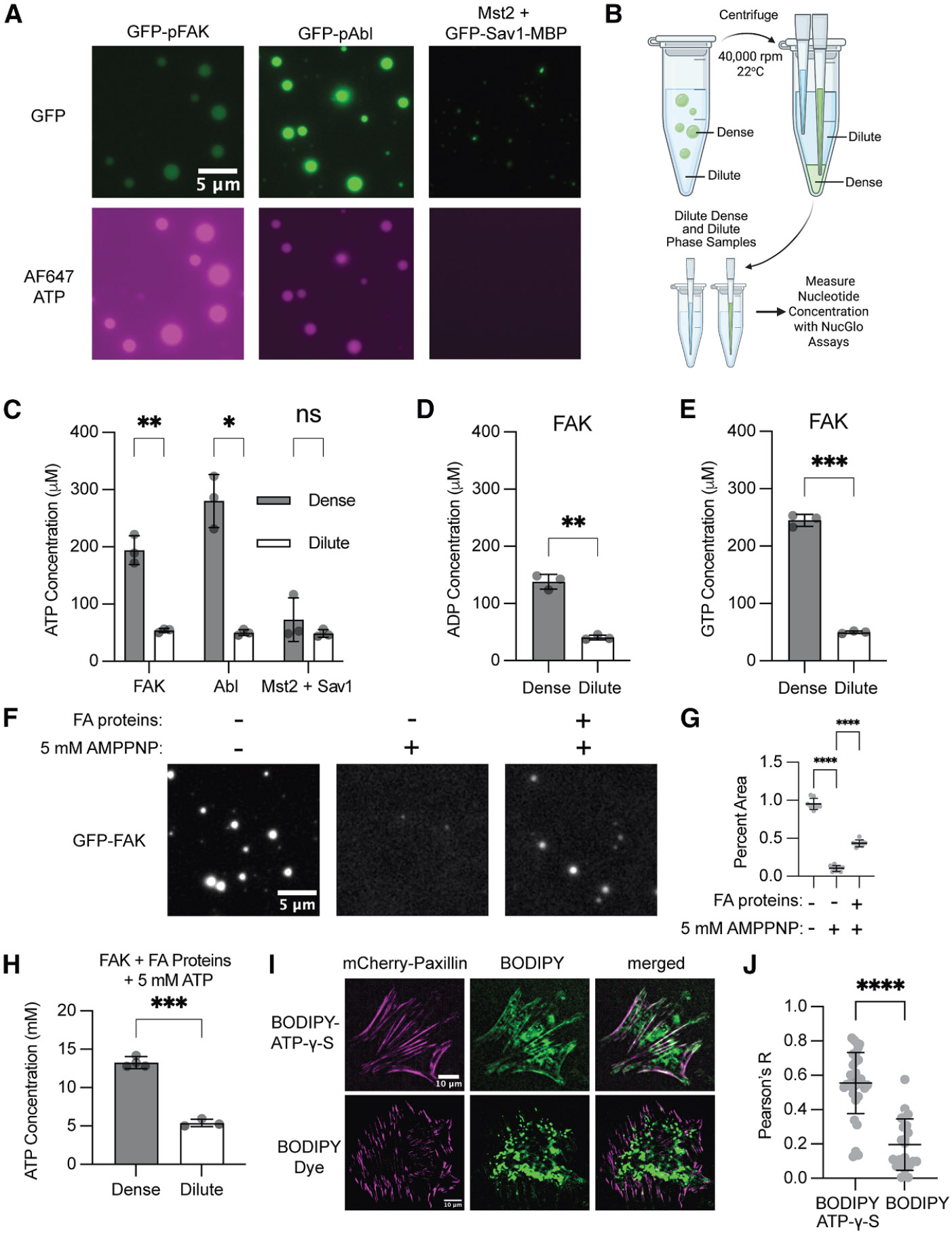
FAK and Abl condensates enrich ATP independent of active site binding (A) Images of 1 μM mEGFP-FAK, 1 μM mEGFP-Abl, or 100 nM mEGFP-Sav1-MBP +100 nM Mst2 with 2.5 μM AF647-ATP. Scale bars, 5 μm. (B) Diagram of sedimentation-luciferase assays for nucleotide measurements. (C–E) Measurements of ATP (C), ADP (D), and GTP (E) concentrations in the dense and dilute phase with 50 μM total nucleotide. (F) Images of 1 μM mEGFP-FAK. “FA proteins” denotes the addition of the purified focal adhesion proteins paxillin, p130Cas, Nck and N-WASP at 1 μM. Scale bars, 5 μm. (G) Quantification of percent area occupied by condensates in (F). n = 8 images. Significance tested with Brown-Forsythe and Welch ANOVA with Dunnett’s T3 multiple comparison tests. (H) Measurements of ATP concentration in the dense and dilute phase with 5 mM total ATP. (I) Representative TIRF microscopy images of MEFs. Scale bars, 10 μm. (J) Pearson’s correlation coefficient between focal adhesions (mCherry-paxillin marker) and BODIPY-ATP-γ-S or BODIPY dye alone (n = 35 and 23 cells, respectively). For (C), (D), (E), (H), and (J) significance was tested with unpaired *t* tests with Welch’s correction. For all sedimentation-luciferase assays, n = 3 replicates. For all *in vitro* experiments, buffer conditions match those in Figure 1B FAK and Figure 3B (Abl and Sav1+Mst2). For all graphs error bars represent standard deviation and *p < 0.0332, **p < 0.0021, ***p < 0.0002, ****p < 0.0001. Also see Figures S11-S15.

In addition to concentrating proteins, condensates create unique environments with emergent chemical and physical properties.^7^ For example, condensates create distinct chemical environments that can enrich or exclude specific small molecules and metabolites.^41,42^ Since ATP is a critical substrate for kinases, we determined the extent to which ATP is enriched or excluded from reconstituted kinase condensates. At micromolar concentrations, a fluorescent analog of ATP (Alexa 647-ATP) is enriched within FAK and Abl condensates but neither enriched nor excluded from Mst2 condensates (Figure 4A). The free Alexa 647 dye does not show enrichment or exclusion from these condensates and BODIPY-ATP-γ-S is similarly enriched in FAK condensates, indicating that ATP is likely driving this enrichment (Figures S11A and S11B). Additionally, AF647-ATP rapidly enters mEGFP-FAK condensates (t_1/2_ = 2.2 ± 1.2 s) (Figure S11C). Since fluorescent dyes can alter the chemical properties of nucleotides and often exhibit different fluorescent properties depending on chemical environment, we developed a luciferase-based assay to directly quantify unmodified nucleotide concentrations in the dense and dilute phases (Figure 4B). We validated this assay by comparing to spectrophotometric measurements of nucleotide concentrations in poly-lysine condensates (Figure S12).^43^ When we added 50 μM ATP to experiments with kinase condensates, the dense phase ATP concentrations were significantly higher than the dilute phase for FAK and Abl condensates (194 μM and 280 μM, respectively) while not significantly different for Mst2:Sav1 condensates (72 μM; Figure 4C). Magnesium was omitted from these experiments to prevent binding of ATP to the kinase active site and to prevent autophosphorylation activity in the samples.^44^ We also found that ADP and GTP were significantly enriched in the dense phase of FAK condensates (Figures 4D and 4E). Since GTP has undetectable binding to the FAK-active site,^45^ we conclude that kinase condensates can enrich nucleotides independent of stereospecific binding to the active site.

Next, we wanted to determine if kinase condensates could enrich ATP at physiological nucleotide concentrations. In cells, ATP is complexed with magnesium and is maintained at millimolar concentrations in 20-1,000-fold excess of ADP.^46^ However, millimolar concentrations of ATP can also reduce or enhance phase separation depending on the context.^43,47,48^ To determine if kinase phase separation was sensitive to ATP independent of kinase ATPase activity, we added 5 mM of AMPPNP, a non-hydrolyzable ATP analog, to *in vitro* condensate assays (Figure S13). We found that Mst2 was unaffected by AMPPNP, Abl formed more condensates with AMPPNP, and FAK formed very few condensates with AMPPNP (Figures S13A and S13B). Combining FAK with additional adapter proteins to form more physiological focal adhesion condensates^26^ partially rescued condensate formation in millimolar AMPPNP (Figures 4F and 4G). Next, we used the luciferase assay to measure ATP enrichment in focal adhesion condensates. When we added 5 mM ATP to focal adhesion condensates, the dense phase contained 13.3 mM ATP (Figure 4H). The ATP concentration in the dense phase was not altered by including 5 μM ADP in the experiment (Figure S14). We also used microscopy to assess ATP enrichment in kinase condensates at millimolar concentrations (Figure S15). When we used buffer containing 1 mM AMPPNP and 10 μM Alexa 647-ATP, we found that focal adhesion condensates and Abl condensates enriched ATP even with 1 mM MgCl_2_ and 50 μM ADP (Figure S15A). Mst2 condensates did not enrich ATP in any conditions. When we used 1 mM ATP instead of AMPPNP, we saw similar Alexa 647-ATP enrichment in focal adhesion condensates across all conditions, but less enrichment in Abl condensates with 1 mM MgCl_2_ (Figure S15B). Including 1 mM ATP-Mg supports rapid autophosphorylation of both Abl and Mst2 (Figures S10D-S10G), and these data suggest that the negative charge added by autophosphorylation reduces ATP enrichment in Abl condensates. Together, these data demonstrate that focal adhesion condensates and Abl condensates enrich ATP at physiological nucleotide concentrations.

To further explore whether ATP enrichment into kinase condensates could be physiologically relevant, we tested the ability of cellular focal adhesions to concentrate BODIPY-ATP-γ-S. Using a previously developed assay to introduce molecules into cells,^49^ we gently permeabilized cells and incubated with BODIPY-ATP-γ-S or BODIPY. We found that BODIPY-ATP-γ-S was non-uniform in its localization in the cytoplasm and nucleus and appeared to enrich in focal adhesions (Figure 4I). Compared to BODIPY, BODIPY-ATP-γ-S was significantly more colocalized with the focal adhesion marker paxillin (Figures 4I and 4J). We conclude that ATP can enrich within both reconstituted focal adhesion condensates and cellular focal adhesions.

### Positive charge drives ATP enrichment into condensates

**Figure 5.**
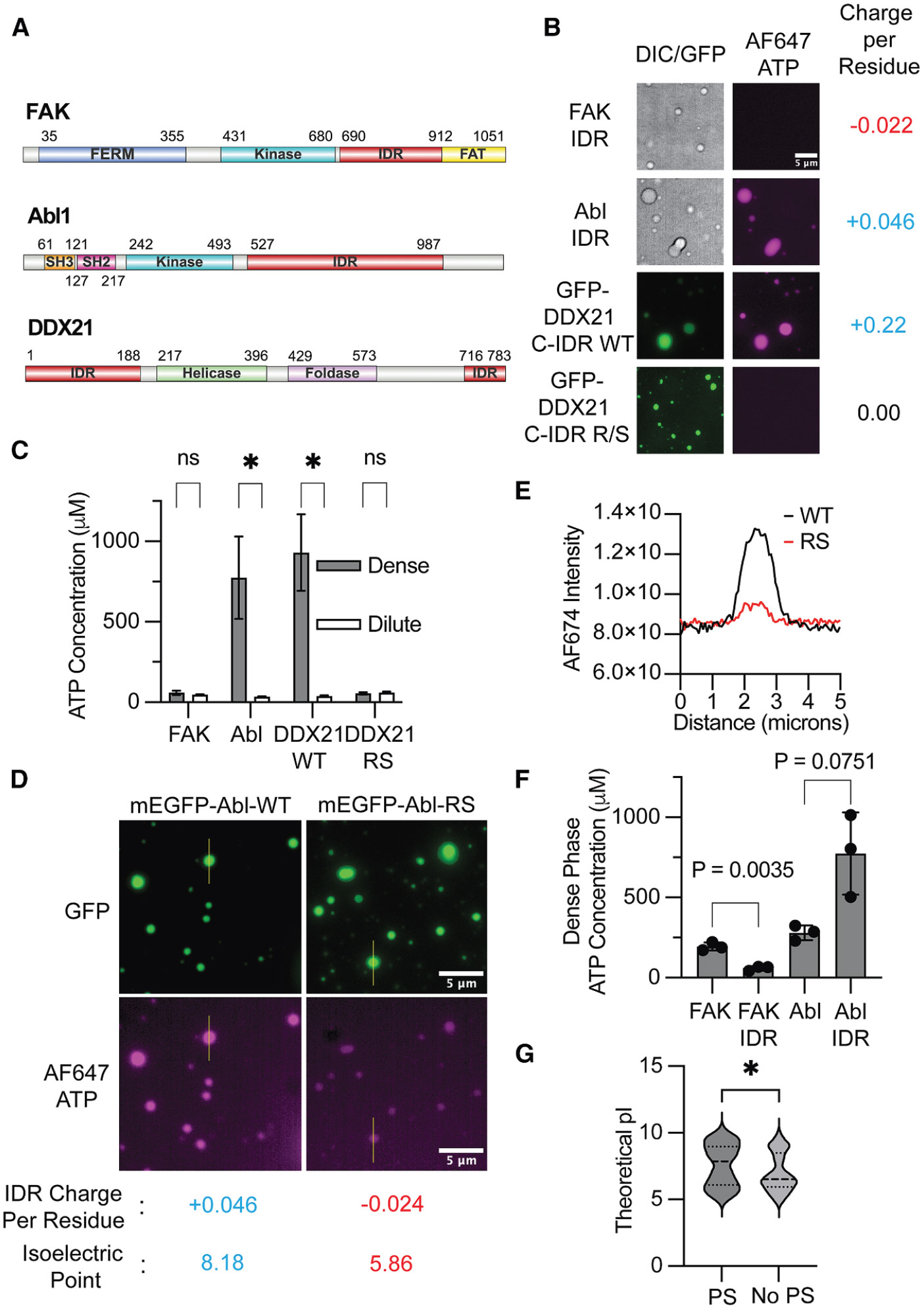
Positive charge is sufficient to enrich ATP in IDR condensates (A) Diagram of FAK, Abl1 and DDX21 proteins indicating intrinsically disordered regions (IDR). (B) Images of IDR condensates with AF647-ATP. Buffer conditions are 20 μM protein, 25 mM HEPES pH 7.5, 100 mM NaCl, 20% PEG8000, 1 mM DTT and 2.5 μM AF647-ATP. (C) Measurements of ATP concentrations in dense and dilute phase from sedimentation-luciferase assays. Buffer conditions are identical to those in (B) except with 50 μM ATP instead of AF647 ATP (n = 3 replicates). (D) Representative fluorescent microscopy images of full-length mEGFP-Abl condensates with AF647 ATP. Buffer is 50 mM HEPES pH7.5, 50 mM NaCl, 1% glycerol, 1 mM DTT and 2.5 μM AF647-ATP. (E) Analysis of linescans (yellow lines) in (D). (F) C**omparison of dense phase ATP concentrations between full length protein condensates and IDR condensates. Data are replotted from (C) and Figure 4C. For (C) and (F), error bars denote standard deviation and statistical tests are unpaired *t* tests with Welch correction.** (G) D**istribution of theoretical isoelectric points (pI) of human protein kinases binned by those predicted (PS, *N* = 68) and those not predicted (No PS, *N* = 94) to phase separate. Statistical test is a Mann-Whitney test. Dotted lines represent quartiles.** For all images scale bars are 5 μm. For all graphs *p < 0.0332, **p < 0.0021, ***p < 0.0002, ****p < 0.0001. Also see Figure S16.

FAK and Abl condensates both enrich ATP, and both kinases contain IDRs (Figure 5A). Previous studies have found that ATP is highly enriched in condensates formed from positively charged IDRs.^43,48,50^ Thus, we hypothesized that positive charge within IDRs could be important for ATP partitioning into kinase condensates. FAK has a negatively charged ~220 amino acid IDR (net charge per residue = − 0.02), while Abl has a positively charged ~460 amino acid IDR (net charge per residue = +0.05) (Figure 5A). Both IDRs form condensates in the presence of crowding agent (20% PEG8000), but only Abl IDR condensates enrich Alexa 647-ATP (Figure 5B). When we added 50 μM ATP, the Abl IDR dense phase enriches ATP 20-fold (773 μM in the dense phase; 32 μM in the dilute phase), but the FAK IDR dense phase does not significantly enrich ATP (59 μM in the dense phase; 45 μM in the dilute phase) (Figure 5C). To further test if positive charge is sufficient to drive ATP enrichment, we investigated the C-terminal IDR of DDX21. DDX21 is a DEAD-box ATPase with a well-characterized positively charged C-terminal IDR (net charge per residue = +0.22) that phase separates.^51^ The DDX21 IDR dense phase enriches ATP 20-fold (931 μM in the dense phase; 37 μM in the dilute phase), and neutralizing the IDR charge is sufficient to abolish ATP enrichment (54 μM in the dense phase; 58 μM in the dilute phase; Figures 5B and 5C). Additionally, Abl and DDX21 IDR condensates enrich ATP when we added 3 mM ATP (5.8-fold ATP enrichment for Abl IDR; 15-fold ATP enrichment for DDX21 IDR; 2.6-fold ATP enrichment for DDX21 R/S IDR; Figure S16A and S16B). We conclude that positive charge is sufficient to enrich ATP within IDR condensates. To test if the positive charge in the Abl IDR is necessary to enrich ATP into full length Abl condensates, we mutated the 33 arginine residues within the IDR to serine. The mutant Abl has a negatively charged IDR (net charge per residue = − 0.024) and an isoelectric point of 5.86 (compared to 8.18 for WT Abl). Condensates formed from the mutant Abl had reduced ATP enrichment compared to WT Abl condensates (Figures 5D and 5E). Thus, positively charged arginine residues within the Abl IDR are directly responsible for ATP enrichment into Abl condensates. Together, these data demonstrate that condensate electrostatic potential is an emergent condensate property that influences nucleotide partitioning.

### Folded domains contribute to ATP enrichment in kinase condensates

Over 80% of human kinases contain both folded domains and IDRs.^52^ Although FAK has a negatively charged IDR that is not sufficient to enrich ATP, the full-length FAK protein forms condensates that enrich ATP (Figure 5F). Additionally, full-length Abl forms condensates that enrich less ATP than the Abl IDR condensates (Figure 5F). This demonstrates that including folded domains with IDRs influences ATP enrichment into condensates. Simulations have shown that ATP can cluster around folded domains through electrostatic interactions with surface exposed basic residues.^53^ In addition to electrostatic potential, other physicochemical properties, such as hydrophobicity, can promote small molecule enrichment into condensates.^41^ Thus, the properties of both IDRs and folded domains influence the emergent condensate physicochemical environment. Since kinase condensates enrich ATP at least in part due to positively charged residues, we computed the theoretical isoelectric point of human kinases. We found that kinases predicted to phase separate have significantly higher isoelectric points compared with kinases predicted to not phase separate (Figure 5G), consistent with a functional role for positive charge within kinase condensates. This suggests that ATP enrichment may be important for kinase signaling in some cellular contexts and/or that ATP may help drive phase separation of some kinases with basic IDRs. Together, our experimental data demonstrate that substrates can be enriched through the emergent chemical properties of enzyme condensates.

## DISCUSSION

Many human kinases can be activated through concentration-dependent autophosphorylation mechanisms.^23^ Our data demonstrate that phase separation is a robust mechanism to accelerate kinase autophosphorylation reactions by increasing kinase concentration. Using *in vitro* kinase assays, we found that condensates elicit rapid and robust autophosphorylation of cytosolic tyrosine (FAK and Abl) and serine/threonine (Mst2) kinases. For the kinases we studied, these autophosphorylation sites included not only activation loop sites, which increase the intrinsic catalytic efficiency of the kinase domain, but also key regulatory sites that release intramolecular inhibitory interactions (Abl) or create binding sites for additional kinases (FAK). This demonstrates that condensates promote autophosphorylation of kinases with diverse structural mechanisms of regulation. Although many specific mechanisms of kinase activation are well established and do not require phase separation, locally enriching kinases in condensates is a general mechanism that could promote concentration-dependent autophosphorylation for many diverse kinases. FAK, Abl, and Mst2 by themselves did not exhibit significant changes in phase separation upon autophosphorylation, but for other kinases autophosphorylation could dynamically regulate phase separation. For example, autophosphorylation of receptor tyrosine kinases can promote phase separation by creating multivalent binding sites for SH2-domain containing adapter proteins.^15,16^ Thus, condensate formation can trigger autophosphorylation, but autophosphorylation could also feedback to modulate condensate formation or composition.

Kinase phase separation is dependent on both kinase concentration and solution conditions, such as temperature, pH, salt, and molecular crowding.^31^ Our findings suggest that kinase phase separation could directly activate numerous kinase signaling pathways in response to changes in cellular conditions that promote phase separation.^6^ For example, WNK is a kinase that can be activated by *trans*-autophosphorylation of its activation loop,^54^ and WNK phase separates upon molecular crowding during hypertonic stress. WNK condensate formation leads to signaling activation, consistent with condensates triggering *trans*-autophosphorylation.^11^ Our data also reveal how kinase condensates can potentially dysregulate signaling pathways. We found that kinase overexpression is sufficient to form cytoplasmic condensates that decouple autophosphorylation from upstream receptor activation. Kinase autophosphorylation is not sufficient for full signaling pathway activation, and FAK cytoplasmic condensates may not fully rescue normal integrin-dependent signaling. FAK and Abl are both overexpressed in certain cancers, and kinase overexpression could trigger aberrant condensate formation to constitutively autophosphorylate kinases.^55,56^ Thus, gene fusions that create chimeric fusion kinases^14,17-19^ and overexpression of endogenous kinases may both result in kinase condensates that dysregulate signaling and decouple kinase autophosphorylation from normal stimuli.

Condensates do not simply concentrate proteins but also create local chemical environments distinct from the dilute phase. We found that the emergent electrostatic potential of kinase condensates can drive nucleotide enrichment independent of stereospecific high-affinity binding. This enrichment is driven, in part, by positively charged IDRs. We found that arginines within the Abl IDR are necessary for ATP enrichment into full-length Abl condensates (Figures 5D and 5E), but we do not know if these arginines alone are sufficient. Charge patterning within the IDR or arginine-containing motifs could potentially help enrich ATP within condensates. Additionally, we found that the FAK IDR is not sufficient to enrich ATP, suggesting that folded domains can also contribute to ATP enrichment (Figure 5F). Positively charged IDRs and folded domains function together to create an internal condensate electrostatic potential that favors enrichment of negatively charged nucleotides.

Enrichment of charged small molecules due to the emergent electrostatic potential of protein condensates has been described before both in synthetic systems and natural condensates.^48,57^ Moreover, small molecules have been shown to enrich into condensates through high affinity stereospecific binding to folded domains.^14^ Unlike these earlier observations, kinase condensates enrich an essential substrate through condensate electrostatic potential. In addition to influencing ATP enrichment, positively charged IDRs can also promote phase separation through interactions with ATP. We observe that Abl, which has a positively charged IDR, phase separates more with millimolar concentrations of AMPPNP. Kinases predicted to phase separate tend to have higher isoelectric points, consistent with a functional role for positive charge within kinase condensates. Moreover, extensive phosphorylation can dramatically reduce positive charge and would thus be expected to reduce ATP partitioning. This suggests that the dynamic modulation of protein charge through phosphorylation can negatively feedback to tune ATP enrichment and kinase activity inside condensates. Maintaining a high ATP concentration in condensates could help drive phosphorylation in normal cell processes and potentially sustain kinase activity in cellular conditions where free ATP concentrations are limiting.

### Limitations of the study

We initiate all the *in vitro* phosphorylation assays by adding ATP to the sample. Comparing the autophosphorylation rates between dilute and total solutions is potentially confounded by the additional time it takes ATP to enter the condensates. AF647-ATP enters mEGFP-FAK condensates quite rapidly (t_1/2_ = 2 s) and our initial phosphorylation time point is measured at 30 s. If ATP diffusion into condensates was rate limiting, our assays would underestimate the actual phosphorylation rate in the dense phase.

In Figure 1, we relied on a phospho-specific antibody to quantify FAK autophosphorylation. Although antibodies have the advantage of reporting phosphorylation rates of a specific residue (Y397 of FAK), western blots are not as sensitive as luminescent or colorimetric assays. We rigorously establish linearity of our western blot standards in both channels (pan-FAK and pY397 FAK) to enable accurate relative comparisons of Y397 phosphorylation between samples. While the overall dynamic range of this assay is limited, we are confident in our conservative estimates of the activity increase between 30 nM and 60 nM. We confirmed the general effect of increased activity in FAK condensates (Figure 1H) with a more sensitive luminescence assay (Figure S10C), but a more sensitive luminescence or colorimetric assay could more accurately measure the fold-increase achieved by crossing the C_dilute_.

We use an overexpression system to induce FAK condensates in the cytoplasm. While this allowed us to test that condensate formation is sufficient for autophosphorylation, we did not determine whether FAK phase separation is required for normal activation of endogenous FAK at focal adhesions. For our cell assays, we used poly-D-Lysine as a nonspecific substrate to anchor cells to the imaging surface without initiating integrin-dependent FAK signaling. We found that the fibroblasts initiate integrin-dependent signaling within 60 min of plating. Thus, we are unable to assess integrin-independent signaling from induced cytoplasmic FAK condensates at time points longer than 30 min.

Finally, our study focused on kinase autophosphorylation, but how these condensates affect the phosphorylation rate or specificity of downstream substrates was not assessed. Although kinase condensates increase autophosphorylation, which is expected to activate kinases, we cannot, and should not, conclude that kinase condensates will universally enhance signaling in a cellular context. Kinase condensates must be studied in specific cellular contexts to determine their effect on signaling pathway activation.

## STAR★METHODS

### EXPERIMENTAL MODEL AND STUDY PARTICIPANT DETAILS

#### Mouse embryonic fibroblasts

p53 −/− FAK +/+ mouse embryonic fibroblasts (ATCC CRL-2645) were harvested from E8.0 day old mouse embryo and cultured in Dulbecco’s Modified Eagle Medium (DMEM) supplemented with 10% Fetal Bovine Serum (FBS), Penicillin/Streptomycin (100 U/mL and 100 mg/mL, respectively) and Glutamax (2 mM) at 37°C with 5% CO_2_. Cells were routinely tested for mycoplasma and subcultivated at a ratio of 1:8. The sex of these cells is unspecified from the supplier and original source. Cells for experiments were used at low passage numbers and had similar morphology and doubling times to the authenticated cell line from the supplier. Cells were not further authenticated after receipt from the supplier.

#### Bacterial strains

BL21 (DE3), Rosetta^™^ 2 (DE3), NEB^®^ 5-alpha Competent and DH10αEMBacY *E. coli* cells were grown in LB medium supplemented with appropriate antibiotics at 37°C.

#### Insect cells

HighFive^™^ Trichoplusia ni and Sf9/Sf21 *Spodoptera frugiperda* cells were grown in ESF-921 (Expression Systems, 96-001-01) media at 27°C and 100 rpm. Cells were maintained at 1 million cells/mL.

### METHOD DETAILS

#### Cloning

Cloning of plasmids used in this study was done with Gibson Assembly and/or site directed mutagenesis using the Q5^®^ Site-Directed Mutagenesis Kit (NEB). All plasmids were sequenced with whole plasmid sequencing (Plasmidsaurus) before experiments to confirm sequence and absence of point mutations. All plasmids generated in this study and their sequences will be submitted to Addgene.

#### Baculovirus generation and protein expression

300 ng of pFAST plasmids containing proteins of interest were electroporated into DH10αEMBacY *E. coli* and grown at 37°C and 200 rpm for 4 hours. Cells were then plated on XGal+IPTG plates and incubated at 37°C overnight. White colonies were inoculated into 5 mL of LB + Gentamicin and grown overnight at 37°C and 200 rpm. Cultures were centrifuged at 4000 rpm for 10 minutes and cell pellet was resuspended in 50 mM Tris pH 8.0, 10 mM EDTA, 50 mM Glucose, 0.1 mg/mL RNAse A. Cells were lysed with 250 μL of 0.2 M NaOH and 0.1% SDS and gently inverting 3-5 times. The solution was then immediately neutralized with 350 μL of 4M KoAc pH 5.5 and inverting 3-5 times. This solution was centrifuged at 15,000 rpm for 10 minutes and supernatant was collected and centrifuged again to remove any cell debris. 700 μL of isopropanol was added to the supernatant and incubated at −80°C for 1 hour. After this, the solution was thawed and centrifuged at 15,000 rpm for 30 minutes at 4°C. Supernatant was discarded and 500 μL of 70% ethanol was added and sample was centrifuged at 15,000 rpm for 10 minutes at 4°C. Supernatant was discarded and the bacmid DNA pellet was covered with 30 μL 70% ethanol and stored at −20°C until used for transfection. Ethanol was removed and bacmid DNA was air dried for 5 minutes before adding 20 μL of water to dissolve the bacmid DNA. Care was taken to not pipette the bacmid DNA solution to preserve supercoiling.

3 μL of bacmid DNA was transfected into 1 million Sf9 cells plated on a culture treated plate using X-tremeGENE^™^ 9 DNA Transfection Reagent (Millipore Sigma) following the manufacturer’s protocol. Cells were incubated for 3-5 days and transfection and expression efficiency was determined by microscopy of YFP expressing cells. The supernatant was collected and added to 25 mL of Sf21 cells grown in suspension at 100 rpm and 27°C. After 3-5 days cells were centrifuged at 238 g for 15 minutes and the supernatant was collected as the baculovirus stock and stored at 4°C. For all proteins expressed from baculovirus, 2 - 5 mL of this stock, depending on virus titer and age, was added to 600 mL of HighFive^™^ Trichoplusia ni cells grown in suspension at 100 rpm and 27°C. After 2 – 4 days, cells were harvested by centrifuging at 238 g for 30 minutes at 4°C.

#### Microscopy

TIRF images were captured using LASX acquisition software to drive a Leica TIRF-module mounted on a Leica DMi8 with DIC optics equipped with a plan apo 100 Å~1.47 NA TIRF objective and a Quad Band set filter cube for TIRF applications. Illumination was provided by an integrated laser system equipped with multiple laser lines (405 nm-50mw/488 nm-150mw/561 nm-120mw/638 nm-150mW). Images were acquired using a Hamamatsu Flash 4.0 V3 CMOS camera.

Epifluorescence and DIC images were captured using LASX acquisition software to drive a Leica DMi8 with DIC optics equipped with a plan apo 100 Å~1.47 NA TIRF objective. Illumination was provided by an LED8 light source equipped with two interchangeable filter cubes (one for excitation at 391/32, 479/33, 554/24, 638/31; one for excitation at 473/22, 539/24, 641/78, 810/80). Images were acquired using a Hamamatsu Flash 4.0 V3 CMOS camera.

Spinning disk confocal images were captured using MetaMorph acquisition software to drive a Zeiss AxioVert 200M inverted microscope stand with DIC optics equipped with a Yokogawa CSU-22 spinning disk confocal scan head with Andor Borealis modification. Illumination was provided by an Andor integrated laser engine equipped with multiple laser lines (405nm, 445nm, 488nm, 515nm, 561nm, and 642nm). Images were acquired using a Hamamatsu Orca-ER cooled CCD camera.

#### Preparing PEG-Silane coated plates

384-well glass-bottom plate (Cellvis, P384-1.5H-N) was washed with 5% Hellmanex III (Hëlma Analytics) for 3.5 hrs at 55°C and thoroughly rinsed with MilliQ H_2_O. Plates were then washed with 1 M NaOH for 1 hr at 55°C and thoroughly rinsed with MilliQ H_2_O. 50 μ L of 20 mg/mL mPEG silane MW 5 k (Creative PEGworks) in 95% EtOH was added to each well. The plate was covered in parafilm and incubated overnight at room temperature. The plate was thoroughly rinsed with MilliQ H_2_O, dried, and sealed with foil.

#### Microscopy of condensates

Representative images of *in vitro* condensates were taken with epifluorescence or differential interference contrast (DIC) microscopy. The buffer conditions used for specific experiments are denoted in figure legends. For all experiments, stock proteins were thawed quickly at room temperature then spun at 21,000 rpm at 4°C for 10 minutes to remove any aggregates. The supernatant was transferred to a new tube and A280 values were measured using the protein storage buffer as a blank. Protein concentrations were calculated based on predicted extinction coefficients. If necessary, protein stocks were diluted in their respective storage buffer before being diluted into an experimental buffer that yielded the desired final concentrations of all buffer components (buffer, salt, etc.) after accounting for contributions from the protein storage buffers. For all experiments, protein was always added after buffer with the protein/s required for phase separation added last. The solution was mixed by pipetting up and down before loading solutions into a 384-well PEG-silane coated glass-bottom plate. Samples were incubated for 20-30 minutes before imaging. For each experiment, regions of interest were randomly selected for imaging, and all images were acquired within 10 minutes of each other. Fluorescence microscopy images were analyzed in ImageJ^58^ to calculate the percent droplet area. The background was subtracted with a rolling circle of radius 25 pixels, then hot/dead pixels were removed by using a median filter with pixel size=2. Then any fields of view (FOV) with large auto-fluorescent debris were removed. Images were auto-thresholded with the Li algorithm and “Analyze Particles” was run to calculate percent area covered by droplets for each image. For DIC microscopy analysis droplets were thresholded by training a Pixel Classification segmentation algorithm in Illastik on one image to be analyzed then used to segment the remaining images. “Analyze Particles” was run in ImageJ to calculate percent area covered by droplets for each image. For all microscopy experiments, representative images for figures were selected such that the percent area of the representative image closely matched the average of all the images for that condition.

#### Turbidity measurements

50 μL phase separated solutions were prepared as described for the microcopy experiments and incubated for 45 minutes. After incubation, samples were gently mixed by pipetting 3-4 times with a 200 μL pipette tip and then carefully transferred into a low volume quartz cuvette. Absorbance at 350 nM was measured, and the final buffer without protein was used as the blank measurement.

#### FAK phase separation autophosphorylation assays

FAK proteins were incubated in 50 mM HEPES pH 7.5, 50 or 300 mM NaCl (low or high salt conditions), 250 μM MnCl_2_, 0.435% (v/v) glycerol, and 1 mM DTT. A 100 μL mixture was made and 15 μL of this mixture was quickly aliquoted into 7 PCR tubes, one for each timepoint. Mixtures were incubated for 45 minutes at room temperature to reach equilibrium. 0.75 μL of a 100 mM stock of ATP in 100 mM Tris-HCl pH 7.5 was added to 999.25 μL of 25 mM HEPES pH 7.5, 50mM NaCl, 1 mM DTT to yield a 75 μM ATP solution. To initiate the reactions, 2 μL of the 75 μM ATP solution was added to the FAK mixture and instantly mixed 3-4 times by pipetting up and down with a 10 μL pipette tip. The reactions were quenched by adding 4.5 μL of Quench Buffer (150 g/L SDS, 0.3 M Tris pH 6.8, 25% (v/v) glycerol) preheated to 99°C and instantly mixed 3-4 times by pipetting up and down with a 10 μL pipette tip. Reactions were then incubated at 99°C for 5 minutes, then placed on ice. Reactions were spun down in a tabletop centrifuge to collect all the samples to the bottom of the tube and 5 μL was diluted with 95 μL of 2X SDS Loading Buffer (100 mM Tris pH 6.8, 10% (v/v) 2-mercaptoethanol, 4% (w/v) SDS, 0.2% (w/v) Bromophenol Blue, 20% (v/v) glycerol). This dilution was determined to load samples in the middle of the linear range of both the pan-FAK and pY397 FAK channels for the western blot protocol. Samples were analyzed by western blot. Primary antibodies were mouse Anti-FAK, clone 4.47 (Sigma; 1:2000) and rabbit Anti-Phospho-FAK (Tyr397) (Invitrogen; 1:2000). Secondary antibodies were goat Anti-mouse IgG (H+L) (DyLight^™^ 680 Conjugate; 1:10,000) (Cell Signaling Technology, #5470) and goat Anti-rabbit IgG (H+L) (DyLight^™^ 800 4X PEG Conjugate: 1:10,000) (Cell Signaling Technology, #5151).

All blots were analyzed in ImageJ using the same region of interest (ROI) size and shape. This ROI was moved to surround each band in the pan-FAK channel and pasted onto the same position in the pY397 channel to measure the average intensity of the same band in both channels. Background measurements were taken from areas just below or above each band. For each band, its associated background measurement was subtracted from the average intensity. Finally, the pY397 value was divided by the pan-FAK value for each timepoint to generate the normalized pY397 signal in Figure 1E.

#### FAK total vs dilute autophosphorylation assays

Assays were performed as described above in “FAK Phase Separation Autophosphorylation Assays” with the following modifications. A 200 μL solution of 1 μM dephosphorylated FAK in a final buffer of 50 mM HEPES pH 7.5, 50 mM NaCl, 250 μM MnCl_2_, 1% glycerol, 1 mM DTT was made in a 1 mL ultracentrifuge tube (Beckman, #347356). This was immediately mixed, and half was removed to a separate ultracentrifuge tube. These were incubated or 1 hour at room temperature to reach equilibrium. One tube (dilute sample) was then spun down at 40,000 rpm at 22°C for 3 hours to remove the dense phase. 50 μL of the supernatant was removed as dilute phase. The tube that was not centrifuged was incubated stationary at room temperature then mixed by pipetting up and down gently 4-5 times to resuspend any settled droplets. Reactions were run as described above for 30 second timepoints and 0 second timepoints with no ATP added. Quenched samples were diluted appropriately with 2X SDS Loading Buffer (100 mM Tris pH 6.8, 10% (v/v) 2-mercapto-ethanol, 4% (w/v) SDS, 0.2% (w/v) Bromophenol Blue, 20% (v/v) glycerol) to a final concentration of ~7 nM FAK (3.025-fold dilution of dilute samples and 100-fold dilution of total samples). Samples were run along with 6 phosphorylated FAK standards. These standards were made from purified phosphorylated FAK diluted in protein storage buffer. The final concentrations of the standards were (before dilution in 2X SDS Loading Buffer) 0.977 nM, 1.953 nM, 3.906 nM, 7.8125 nM, 15.625 nM, and 31.25 nM. Samples were analyzed by western blot using the antibodies described above.

For all bands measurement and background subtraction was performed as described in “FAK Phase Separation Autophosphorylation Assays”. The FAK and pFAK measurements of the standards were plotted as amount loaded in femtomoles vs average intensity. These plots were fit with a linear regression, and the equation of this line was used to generate absolute amounts of FAK and pY397 for each sample. The pY397 FAK amount was normalized by dividing by the FAK amount for each sample. The resulting value of the 0 second timepoint was subtracted from the 30 second timepoint and this was divided by 30 seconds to yield the rate. The values were then multiplied by their respective dilution factors (100 for total samples and 3.025 for dilute samples). These values were then multiplied by 100 to generate arbitrary units for ease of displaying the data.

#### FAK titration autophosphorylation assays

Assays were performed as above in “FAK Phase Separation Autophosphorylation Assays” with the following modifications. 25 μL solutions of dephosphorylated GFP-FAK at indicated concentrations (10-80 nM) were made in a final buffer of 25 mM HEPES pH 7.5, 50 mM NaCl, 1% glycerol, 1 mM DTT in Low Protein Binding Microcentrifuge Tubes (Thermo) and incubated for 2 hours at room temperature. 2.5 μL of a 25 mM HEPES pH 7.5, 50 mM NaCl, 1 mM DTT, 1.1 mM ATP, 2.75 mM MnCl2 solution was added to initiate reactions. ATP wasn’t added to one sample to indicate starting phosphorylation amount. Reactions were incubated at room temperature for 30 seconds. Reactions were quenched with 6.88 μL of Quench Buffer. Quenched samples were diluted appropriately with 2X SDS Loading Buffer (100 mM Tris pH 6.8, 10% (v/v) 2-mercapto-ethanol, 4% (w/v) SDS, 0.2% (w/v) Bromophenol Blue, 20% (v/v) glycerol) to a final concentration of 3.636 nM mEGFP-FAK. Samples were run along with between 6 and 8 phosphorylated FAK standards. These standards were made from purified FAK that was not dephosphorylated diluted in protein storage buffer. The final concentrations of the standards were (before dilution in 2X SDS Loading Buffer) 0.244 nM, 0.488 nM, 0.977 nM, 1.953 nM, 3.906 nM, 7.8125 nM, 15.625 nM, and 31.25 nM. It was apparent from the resulting blots that these standards were not fully phosphorylated, which precluded absolute quantification of pFAK and FAK amounts. The standards, however were both linear in both channels throughout the concentrations loaded, and all samples were in this linear range, allowing accurate relative comparisons of autophosphorylation activity between samples.

For all bands, measurement, background subtraction, standard curve generation, and pY397 normalization to total FAK was performed as described in “FAK Total vs Dilute Autophosphorylation Assays” above. These values were then multiplied by their respective dilution factors to yield the plotted values.

#### Western blotting

10 μL of each sample was loaded on a 10% Tris/Glycine SDS-PAGE gel and run at 240V for 30 minutes. 5 μL of a 1:100 dilution of Color Prestained Protein Standard, Broad Range (10-250 kDa) (NEB) in 2X SDS Loading Buffer was loaded as well. Additionally, for autophosphorylations assays a control sample with 2 μL of water added in lieu of the ATP solution was run to show the phosphorylation state of the starting protein. SDS-PAGE gels were washed quickly in TBS then assembled into transfer sandwich with a Low Fluorescence PVDF membrane pre-wet in 100% methanol and 5 pieces of Whatman paper (per side) pre-soaked in 1X Trans-Blot Transfer buffer (BIO-RAD). Transfer was run in a Trans-Blot Turbo (BIO-RAD) with MIXED setting. Membranes were then washed quickly in TBS then transferred to an incubation box with 5 mL of Azure Fluorescent Blot Blocking Buffer (Azure Biosystems). Membranes were incubated at room temperature with gentle shaking for 1 hour to block. Primary antibodies were added at indicated dilutions and incubated at 4°C overnight with gentle shaking. Membranes were washed 3 times with 20 mL Azure Fluorescent Blot Washing Buffer (Azure Biosystems). Membranes were incubated with 5 mL of secondary antibodies in Azure Fluorescent Blot Blocking Buffer at room temperature with gentle agitation for 1 hour. The membrane was transferred to a new incubation box with 20 mL of Azure Fluorescent Blot Washing Buffer and quickly washed. This step was repeated once, then the membrane was transferred to a new incubation box with 20 mL of Azure Fluorescent Blot Washing Buffer and incubated at RT with gentle shaking for 5 minutes. The wash buffer was decanted, and the blot was washed again for 5 mins. This was repeated for 3 total 5-minute washes. The membrane was transferred to a new incubation box with 20 mL of TBS and quickly washed. The membrane was transferred to a new incubation box with 20 mL of TBS and then imaged using a ChemiDoc MP Imaging System (BIO-RAD).

#### FAK dilute and dense phase concentration measurements via microscopy

Concentrations of mEGFP-FAK in both the dilute and dense phases were measured by using a standard curve of dephosphorylated mEGFP-FAK in a high salt buffer that prevents phase separation. These standards were 200 nM, 100 nM, 50 nM, 25 nM and 12.5 nM in High Salt Buffer (25 mM HEPES pH 7.5, 500 mM NaCl, 1 mM DTT, 20 mM glucose, 800 mg/mL glucose oxidase (Sigma, G2133), 140 mg/mL catalase (Sigma, C1345)). For samples to measure the dense phase, 0.1% dephosphorylated GFP-FAK was used at 2 μM in Low Salt Buffer (25 mM HEPES pH 7.5, 50 mM NaCl, 1 mM DTT, 20 mM glucose, 800 mg/mL glucose oxidase (Sigma, G2133), 140 mg/mL catalase (Sigma, C1345)). This 0.1% GFP-FAK was created by mixing the appropriate amount of GFP-FAK and untagged FAK stocks. For samples to measure the dilute phase, 100% dephosphorylated GFP-FAK was used at 50 nM in Low Salt Buffer. These samples, along with the blanks of Low Salt Buffer and High Salt Buffer, were loaded and imaged in wells in a 384-well glass bottom plate that were prepared as described below.

Wells were previously cleaned and coated with PEG-Silane and then incubated with 5 μL of a 1:20,000 dilution of 0.5 μm red FluoSpheres^™^ (Invitrogen^™^) in 100% ethanol for 10 minutes at room temperature until dry. Wells were then incubated with 80 μL of Blocking Buffer (25 mM HEPES pH 7.5, 50 mM NaCl, 1 mM DTT, 1mg/mL BSA) for 30 minutes at room temperature, then washed quickly 3 times with 100 μL of either High Salt Buffer or Low Salt Buffer immediately preceding sample loading. Samples were loaded then incubated for 30-90 minutes while setting up the microscope and imaging of all wells was completed within 2 hours. Wells were imaged with a spinning disc confocal microscope. Red fluorospheres were used to focus the microscope for all measurements.

Standard curves for determining dense and dilute phase concentrations were generated as follows. For each of the standards and the High Salt Buffer blank well, ~10 images of each well were averaged together to create an average image. Any images that showed debris or unusual fluorescence were omitted. The average images of the standards were corrected for uneven illumination by dividing by the average image of the High Salt Buffer sample and then multiplying by the mean pixel intensity of the average image of the High Salt Buffer. Then the mean intensity of each corrected standard was measured, and the mean intensity of the High Salt Buffer (background) was subtracted. These values were fit to a linear regression to create a standard curve. New standards were imaged with each set of experiments.

Dilute phase sample images were corrected for uneven illumination as described above. Images were then thresholded manually to select the regions excluded by the droplets, and the average intensity of this region was measured. The Low Salt Buffer sample was averaged, corrected for uneven illumination, and measured as described above. This background value was subtracted from all of the dilute phase measurements. The resulting background subtracted measurements were converted to dilute phase concentrations using the linear regression of the standard curve.

For dense phase measurements, Z-stacks with width 0.5 mm were taken. The dense phase images were analyzed similarly with the following exceptions. Uneven illumination and background subtraction were performed using the Low Salt Buffer blank. Z-series of each field of view were thresholded for individual 3D droplets using the 3D Object Counter plugin. Threshold settings were determined independently for each replicate. The mean intensity of each 3D droplet was taken to calculate the concentration. Since point spread function and photobleaching affects would lower mean intensity values in the droplets, this is a conservative lower bound estimate of the droplet concentration. Background from the Low Salt Buffer blank was subtracted from these mean intensity values and then the dense phase concentration was calculated from the linear regression of the standard curve. Since these experiments used only 0.1% GFP-FAK (while the standards were made from 100% GFP-FAK) the resulting concentrations were multiplied by 1000 to yield the true dense phase concentration.

#### Mass photometry

Samples of protein were diluted as specified in the figure legends and incubated at room temperature for 2 hours in Low Protein Binding Tubes (Thermo). Buffers were filtered twice using 0.22 μm syringe filters before use. Standards were prepared by diluting NativeMark^™^ Unstained Protein Standard (Invitrogen) 100-fold in identical buffer to samples. Samples and standards were run on the Mass Photometer (Refeyn) using buffer-less focusing. Once samples were loaded, samples were checked for proper focus and adjusted manually and moved to a new region if needed. This procedure only analyzes the dilute phase, since fields of view containing condensates would fail the focus procedure. Contrast values for standards were matched to known molecular weights, and this was used to compute mass values of samples in the Refeyn software.

#### Cell culture and transfection

Mouse embryonic fibroblasts (MEFs, ATCC CRL-2645) were passaged according to the ATCC recommendations. MEFs were cultured in Dulbecco’s Modified Eagle Medium (DMEM) supplemented with 10% Fetal Bovine Serum (FBS), Penicillin/Streptomycin (100 U/mL and 100 μg/mL, respectively) and Glutamax (2 mM) at 37°C with 5% CO_2_. Cells were routinely tested for mycoplasm. All transient transfections were performed with Invitrogen^™^ Lipofectamine^™^ 3000 Transfection Reagent following the manufacturer’s protocol. MEFs were transfected with 8 μg of plasmid DNA at 50% confluency in a 6-well plate. After 24 hours, cells were trypsinized and 50,000 cells were seeded into 8-well iBidi chambers coated with poly-D-Lysine. Cells were imaged by epifluorescence and differential interference contrast (DIC) microscopy at 37°C with 5% CO_2_. Poly-D-Lysine coated plates were prepared with poly-D-Lysine (Thermo Scientific A3890401) following the manufacturer’s protocol. Fibronectin coated plates were made by diluting Bovine Fibronectin (Sigma Aldrich #F1141) 100-fold in Dulbecco’s Phosphate Buffered Saline (DPBS) and incubating wells at 4°C overnight. This solution was removed, and wells were washed once in DPBS immediately before use.

#### Generation of stable DOX-inducible GFP-FAK-WT cell line

MEFs cultured in media with Tet-Free Fetal Bovine Serum were co-transfected with XLone-Puro-mEGFP-FAK-WT (cargo) and Super PiggyBac Transposase plasmid (Gift of Calo lab) in 6-well plates. XLone-Puro-mEGFP-FAK-WT was created by replacing the EGFP with mEGFP-FAK sequence in XLone-Puro-EGFP by Gibson Assembly.^59^ After 3 days (to allow insertion of the cargo and to allow loss of unincorporated cargo plasmid), cells were expanded to 10 cm plates and cells with successful incorporation were selected with 10 μg/mL Puromycin. Control/untransfected cells were also selected for in parallel to assess when selection was successful by noting when all control cells died. After this period (roughly 3-5 days), cells were expanded to 40 million cells, cargo expression was induced by incubating with Doxycycline hyclate for 24 hours, and cells were Flow Assisted Cell Sorted (FACS) sorted. The top 1% GFP expressing cells were collected and expanded to 40 million cells. At this point cells were tested for mycoplasma and aliquots were frozen for storage. For all experiments, mEGFP-FAK-WT was induced with 4 μg/μL of Doxycycline hyclate (Sigma-Aldrich, #D5207) for 24 hours.

#### Fluorescence recovery after photobleaching (FRAP) and FRAP analysis

Puncta/condensates were bleached with exposure to 488 nm laser at 80% power recovery was imaged at timepoints indicated in graphs. FRAP images were analyzed using a region of interest (ROI) outside of the cell as the background signal (BG), an ROI surrounding the entire cell as the total signal (Tot), and an ROI surrounding the mEGFP-FAK puncta as the puncta signal (ROI). Background subtraction, correction for photobleaching and laser intensity fluctuations, and normalization was performed at each timepoint for each puncta to yield the normalized signal at each timepoint using the following equation. The subscript “t” represents the measurement at a given timepoint and the subscript “0” represents the measurement at time zero seconds.


$$Normalized\;Signal_{t}=\frac{ROI_{t}-BG_{t}}{Tot_{t}-BG_{t}}\times \frac{Tot_{0}-BG_{0}}{ROI_{0}-BG_{0}}$$

For each replicate, the intensity of the first timepoint coinciding with the bleach event (I_B_) was subtracted from the normalized signal of all time points, and the resulting data was fit to a single exponential regression to extract t_1/2_ and plateau values. Percent recovery was determined by dividing the plateau value extracted from the regression by (1-I_B_). FRAP analysis of mEGFP-FAK condensates *in vitro* was performed similarly, except using an unbleached droplet as the total ROI to account for photobleaching and laser intensity fluctuations over time.

#### Puncta vs cell intensity analysis

Cells transfected with indicated constructs were plated on poly-D-Lysine as described. 15x15 field of view (FOV, 132x132 micron each) tilescans were imaged with DIC microscopy and epifluorescence microscopy, acquiring Z-stacks of each FOV to capture the entire volume of cells. Maximum intensity projections of each FOV were created, and the FOVs were stitched using LASX software. Individual cells were cropped in ImageJ and autothresholded with Otsu algorithm. Mean fluorescence intensity of each cell was measured using ImageJ “Analyze Particles”. The number of puncta in each cell were counted manually. Cells with large amorphous aggregates were excluded from the puncta analysis (Data on aggregates can be found in Figure S5). The total number of cells in the tilescan (both transfected and untransfected) was estimated by counting cells in the DIC channel, and percent of total cells with puncta was calculated for western blot interpretation. For conditions co-expressing mCherry-Paxillin analysis was limited to cells expressing a small range of mCherry signal (300-600 raw intensity values) which was empirically determined to be the smallest range that still captured a wide range of GFP signals. This keeps the paxillin concentration relatively constant in the analyzed cells, enabling measurement of the specific effect of increasing mEGFP-FAK concentrations.

#### Cell lysate western blots

MEFs were either transfected with 8 μg of plasmid DNA, mock transfected (No DNA), or untreated at 70-80% confluency in a 6-well plate. After 24 hours, all cells were trypsinized and seeded into 6-well plates coated with Poly-D-Lysine for 30 minutes. One sample (ECM) of untreated cells was not trypsinized or re-plated to act as a reference for normal integrin-activated pY397 FAK/pan-FAK (or pCas/Cas or pPax/Pax) ratios. At this point, cells were washed twice in ice-cold phosphate buffered saline (PBS) and lysed with 100 μL of ice-cold RIPA buffer (Thermo Scientific #89900) supplemented with Halt^™^ Protease and Phosphatase Inhibitor Cocktail (ThermoFisher, Cat#78440). Lysates were collected and stored at −20°C until used for western blots. Total protein concentration was measured by diluting a small amount of lysate in 25 mM HEPES pH 7.5 and using a Micro BCA assay (ThermoFisher Scientific #23235) with albumin standards prepared in 25 mM HEPES pH 7.5. Dilutions of the reference sample were run on each western as both adhesion positive pFAK/FAK reference samples and on-blot standards to confirm linearity of pan-FAK and pY397 FAK signals. Samples were loaded accordingly to attain pan-FAK and pFAK signals in this linear range. Samples were diluted 2-fold in 2X SDS Loading Buffer (100 mM Tris pH 6.8, 10% 2-mercapto-ethanol, 4% SDS, 0.2% Bromophenol Blue, 20% glycerol) and boiled at 99°C for 10 minutes, then placed on ice for 5 minutes. 10 μL of each sample was run on a 10% Tris-Glycine gel for 35 minutes at 240V. Transfer, blocking and antibody incubation was performed identically as described earlier for FAK and pY397 FAK. For each blot a linear regression of the standards was used to determine the relative amount of pan-FAK and pY397 FAK in each sample. The pY397 FAK signal was divided by the pan-FAK signal for each sample and then normalized so the values of the reference samples equaled 1. These normalized values represent the percent of reference pY397/FAK ratios recovered by each condition. Also, because of the different mobilities of the GFP tagged FAK and endogenous FAK, it was possible to analyze both species separately on the same blot. For samples that were stained for total protein, this was done with the AzureRed Fluorescent Total Protein Stain (Azure Biosystems, Cat#AC2124) following the manufacturer’s protocol. Lysates were also blotted for pCas/Cas and pPax/Pax analysis following the same protocol except using the following primary antibodies and dilutions; 1:1000 Mouse anti p130Cas (BD Transduction Laboratories^™^, Purified Mouse Anti-p130 [Cas] #610271), 1:1000 Rabbit anti pY410 p130 Cas (Cell Signaling Technology, Phospho-p130 Cas (Tyr410) Antibody #4011), 1:10,000 Mouse anti paxillin (BD Transduction Laboratories^™^, Purified Mouse Anti-Paxillin #612405), 1:1000 Rabbit anti pY118 paxillin (Cell Signaling Technology, Phospho-Paxillin (Tyr118) Antibody #2541).

#### Immunostaining

All immunostaining was performed using the protocol below. The following antibodies and concentrations were used. 1:100 Rabbit anti pY397 FAK (Invitrogen, Phospho-FAK (Tyr397) Polyclonal Antibody #44624G), 1:100 Rabbit anti EEA1 (Cell Signaling Technology, EEA1 Antibody #2411), 1:50 Rabbit anti Rab11 (Cell Signaling Technology, Rab11 (D4F5) XP^®^ Rabbit mAb #5589), 1:250 AF647 Donkey anti Rabbit (Invitrogen, Donkey anti-Rabbit IgG (H+L) Highly Cross-Adsorbed Secondary Antibody, Alexa Fluor^™^ Plus 647, # A32795TR). Exogenously expressed mEGFP-FAK was imaged using the intrinsic GFP fluorescence.

All steps were carried out in 8-well iBidi microplates coated with Poly-D-Lysine. Because cell adhesion under these conditions is weak, care was taken to add and remove buffers slowly on the side of the wells to minimize cell loss. All steps performed at room temperature unless otherwise noted. 50,000 cells were seeded in wells for 10 minutes at 37°C and 5% CO_2_ to allow adhesion to Poly-D-Lysine surface. Cells were then washed 2 times in PBS and fixed with 4% paraformaldehyde (PFA) in tris-buffered saline (TBS) for 15 minutes at 37°C. Cells were then permeabilized with 0.5% (v/v) TritonX-100 in TBS for 8 minutes without shaking. Cells were then washed with 0.1M Glycine in TBS for 10 minutes without shaking. Cells were then washed two times, 5 minutes then 10 minutes, with TBS supplemented with 0.1% (v/v) Tween 20 (TBS-T) with gentle shaking. Cells were then blocked with 2% (w/v) bovine serum albumin (BSA) in TBS-T for 1 hour with gentle shaking. Primary antibodies in 2% BSA in TBS-T were added and cells were incubated for 2 hours with gentle shaking. Cells were then washed three times with TBS-T for 5 minutes. Secondary antibody in 2% BSA in TBS-T were added and cells were incubated for 1 hour with gentle shaking. Cells were then washed three times with TBS-T for 5 minutes. Cells were then quickly washed one time with TBS, the TBS was replaced, and samples were imaged.

#### Mst2 and Abl western blots

To confirm kinase activity in standard kinase buffers, 5 μg of kinase was added to 1X NEBuffer^™^ for Protein Kinases (50 mM Tris-HCl, 10 mM MgCl_2_, 0.1 mM EGTA, 2 mM DTT, 0.01% Brij 35 (pH 7.5 @ 25°C) and 25 μL reactions were initiated with 0.5 mM ATP. Reactions were incubated at room temperature for 2 hours. Mock reactions without ATP added were also set up to assess initial phosphorylation states. Samples were analyzed by western blotting. For MST2 the following primary antibodies were used: 1:1000 rabbit anti Phospho-MST1 (Thr183)/MST2 (Thr180) (Cell Signaling Technology, #49332) and 1:1000 mouse anti STK3 Monoclonal Antibody (4F7) (Abnova). For Abl1 the following antibodies were used: 1:1000 rabbit anti Phospho-c-Abl (Tyr245) Antibody (Cell Signaling Technology, #2861) and 1:1000 mouse anti c-Abl Antibody (24-11): sc-23 (Santa Cruz Biotechnology). Secondary antibodies were goat Anti-mouse IgG (H+L) (DyLight^™^ 680 Conjugate; 1:10,000) (Cell Signaling Technology, #5470) and goat Anti-rabbit IgG (H+L) (DyLight^™^ 800 4X PEG Conjugate: 1:10,000) (Cell Signaling Technology, #5151).

#### ADP-Glo^™^ kinase assays and analysis

Autophosphorylation assays were performed using purified kinases and the ADP-Glo^™^ Kinase Assay kit (Promega #V6930). For all assays, 1 μM protein was incubated at 22°C for 30 minutes to allow condensates to form and reach equilibrium. The buffers for each protein was as follows: mEGFP-dAbl in 25 mM HEPES pH 7.5, 100 mM NaCl, 5% PEG8000, 1% glycerol (v/v), 1 mM DTT, 0.5 mM MgCl_2_, Sav1:dMst2 in 25 mM HEPES pH 7.5, 100 mM NaCl, 10% PEG8000, 1 mM DTT, 0.5 mM MgCl_2_, mEGFP-dFAK in 25 mM HEPES pH 7.5, 50 mM NaCl, 1% glycerol (v/v), 1 mM DTT, 0.5 mM MgCl_2_. Samples were then either centrifuged at 40,000 rpm at 22°C for 1 hour or incubated at 22°C for 1 hour. Supernatant was removed from centrifuged samples to serve as the dilute phase samples. The non-centrifuged samples were used as the total samples. The total samples were mixed by pipetting up and down to resuspend any settled condensates before initiating reactions. Reactions were initiated by adding ATP at a final concentration of 1 μM and immediately mixed. 5 μL samples were removed before adding ATP (0 second timepoint) and every 30 seconds for 2 minutes and mixed with 5 μL of ADP-Glo^™^ Reagent to stop the reaction and deplete unconsumed ATP. These solutions were incubated at RT for 40 minutes. Then, 10 μL of Kinase Detection Reagent was added and incubated at RT for 30 minutes. Luminescence of samples and a 1 μM series of ATP+ADP standards were measured using a BioTek Synergy H1 hybrid reader at room temperature. These ATP+ADP standards were generated according to the manufacturer’s protocol using separate buffers that match each kinases phase separation buffer composition. In short, these standards represent different percentages of ATP conversion to ADP. The kit can only reliably detect down to 1-2% conversion of the total ATP to ADP. The percent of ATP converted to ADP was determined for each sample by using the equation of the linear regression of the ATP+ADP standards. This percentage was then converted to fmol ATP generated. For all samples, the signal of the 0 second timepoint was subtracted from each timepoint and the amount of ADP generated was divided by the seconds of each timepoint. For each sample, the timepoint that led to the largest rate was chosen to represent the intial rate. To be considered to have a detectable signal/rate above the noise the timecourse needed to have at least 3 consecutive points with increasing amounts of ADP generated and the largest signal of those 3 points represent a non-negative amount of ADP generated. If this was not achieved the samples were assumed to be below the limit of detection of the assay.

#### Phospho- mass spectrometry

Samples were prepared identically to the uncentrifuged samples from “ADP-Glo^™^ kinase assays and analysis” as described above. Samples were split and 100 μM ATP was added to one sample to initiate autophosphorylation and incubated at 22°C for 2 hours. The other sample is incubated at 22°C for 2 hours without ATP to serve as the dephosphorylated sample. Samples were digested on S-trap micro spin columns from rotifi. Samples were digested following the manufacturers protocol with the following changes:

Step 5: 10 mM DTT (final concentration) was used instead of TCEP to reduce the proteins and the samples were placed on a heating block for 10 minutes at 95C.

Step 6: 20 mM iodoacetamide (final concentration) was used instead of MMTS, and samples incubate at RT for 30 minutes in the dark.

Step 7: 1% Trifluoroacetic acid (final concentration) was used instead of phosphoric acid.

Step 14: Two separate enzymes were used Trypsin and Chymotrypsin and samples were digested overnight at 37C.

After digestion samples were desalted using Pierce Peptide Desalting Spin Columns (cat#89852) per manufacturer protocol. The tryptic peptides were loaded on a precolumn (Acclaim PepMap 100 75 μM x 2 cm) and separated by reverse phase HPLC (Thermo Ultimate 3000) using a Thermo PepMap RSLC C18 column (2 μm tip, 75 μm x 50 cm PN# ES903) over a gradient (below) before nano-electrospray using a Orbitrap Exploris 480 mass spectrometer (Thermo). Solvent A was 0.1% formic acid in water and solvent B was 0.1% formic acid in acetonitrile.

**Table:** 

Time	% B buffer	Flow rate nL
0	1	200
15	1	200
15.5	3	200
35	23	200
41	35	200
43	80	200
46	80	200
46.1	1	200
60	1	200

The mass spectrometer was operated in a data-dependent mode. The parameters for the full scan MS were: resolution of 60,000 across 375-1500 *m/z* and maximum IT 25 ms. The full MS scan was followed by MS/MS for the top 15 precursor ions in each cycle with a NCE of 28, dynamic exclusion of 10 s and resolution of 30,000. Raw mass spectral data files were searched using Sequest HT in Proteome Discoverer (Thermo). Sequest search parameters were: 10 ppm mass tolerance for precursor ions; 0.02 Da for fragment ion mass tolerance; 2 missed cleavages of trypsin; fixed modification were carbamidomethylation of cysteine and peptide N-termini; variable modifications were methionine oxidation, tyrosine, serine and threonine phosphorylation, methionine loss at the N-terminus of the protein, acetylation of the N-terminus of the protein and also Met-loss plus acetylation of the protein N-terminus. Data was searched against the protein sequences of the kinases used. The canonical autophosphorylation site of Mst2 was not detected due to lack of coverage of that region with chymotrypsin or trypsin digestion.

#### Luciferase assays for measuring nucleotide concentration in dense and dilute phase

250 μL samples of phase separated proteins at concentrations and buffers indicated in figure legends were prepared in 1 mL ultracentrifuge tube (Beckman, #347356). Samples were mixed and incubated for 1 hour at room temperature to reach equilibrium. At this point, nucleotides (ATP, ADP or GTP) were added at indicated final concentrations and mixed. Samples of poly-L-Lysine condensates were prepared similarly except nucleotides were added initially to induce phase separation. Poly-L-Lysine was used at ~100 μM concentration with 5 mM nucleotide in a 10 mM Immidazole pH 7.0 buffer. Poly-L-Lysine was prepared by dissolving poly-L-Lysine hydrochloride powder (Millipore Sigma, Cat# P2658-25MG) in the 10 mM Immidazole pH 7.0 buffer and dialyzing against 1L 10 mM Immidazole pH 7.0 buffer overnight at 4°C to remove chloride ions. The sample was then concentrated to the original volume, aliquoted, flash frozen in liquid nitrogen and stored at −80°C. Samples were then spun down at 40,000 rpm at 22°C for 1 hour to pellet the dense phase. The supernatant was carefully removed and used for dilute phase samples. The dense phase was either collected by repeatedly tapping a pipette tip to force the viscous dense phase into the tip or, when too small to be manipulated this way, all supernatant was removed and dense phase sample was retained in the tube. For dense phase samples that were too small to be manipulated the volume was overestimated to be 1 μL for downstream calculations (which would underestimate concentration calculations later). For samples that could be packed into a pipette tip, the volume was calculated by using a 1 μL mark on the pipette tip as a reference and assuming the pipette tip was a cylinder. Dense phase samples were added to 10 μL of 6M guanidine HCl and mixed to dissolve. Dilute phase samples were diluted 1:10 in 6M guanidine HCl. 5 μL of these samples were then diluted a further 1:100 in 25 mM HEPES pH 7.5, 200 mM NaCl, 10 mM MgCl_2_. Also, experiments with millimolar concentrations of nucleotide required one additional 1:100 (or 1:1000 for poly-L-Lysine dense phase) dilution step in 6M guanidine HCl.

Samples were analyzed with the following luciferase-based kits following the standard protocols: ATP Determination Kit (Invitrogen, A22066), ADP-Glo^™^ Kinase Assay (Promega,V6930), and GTPase-Glo^™^ Assay (Promega, V7681). Standards of nucleotides were made using nucleotides provided in the kits and diluting in the following buffer that matches the final buffer of the samples: 25 mM HEPES pH 7.5, 200 mM NaCl, 10 mM MgCl_2_, 60 mM guanidine HCl. Standard concentrations were 1000 nM, 500 nM, 250 nM, 125 nM, 62.5 nM, 31.25 nM, 15.625 nM, and 7.8125 nM and were run with every replicate. Luminescence of samples was measured with a BioTek Synergy H1 hybrid reader at room temperature. Linear regressions of the standards were used to determine concentrations of samples after accounting for dilution.

This assay was validated by comparing the nucleotide measurements in the poly-L-Lysine dense and dilute phases with nucleotide measurements from absorbance values at 260 nm of the guanidine diluted samples. Since poly-K-Lysine does not absorb at 260 nm significantly at these dilutions, these measurements yield the concentration of nucleotides in the sample. We calculated the concentration of nucleotide from the following extinction coefficients: ATP 15,400 M^−1^cm^−1^, ADP 15,400 M^−1^cm^−1^, GTP 11,700 M^−1^cm^−1^.

#### BODIPY-ATP-γ-S localization in live cells

Cells were transfected with mCherry-paxillin, as previously described. After 24 hours, cells were seeded onto fibronectin coated coverslips for 1.5 hours. Then cells were rinsed with buffer (10 mM MES pH 6.1, 138 mM KCl, 3 mM MgCl2, 2 mM EGTA). Cells were incubated in buffer containing 0.01% Saponin at room temperature for 1 min. Cells were gently rinsed in buffer and incubated with buffer containing 10 μM BODIPY or 10 μM BODIPY-ATP-γ-S for 5 min. Cells were quickly rinsed three times with buffer and imaged immediately. Images were acquired using TIRF microscopy within 5 minutes of rinsing. Pearson’s correlation between mCherry-paxillin image and the BODIPY image was calculated in ImageJ.

#### Phase separation prediction

The phase separation prediction scores (p-scores, 0-1) for human kinases were extracted from three published studies(*32-34*) and compiled into the supplemental spreadsheet (Data S1). PSPire, PSPhunter, and PhaSePred are three computational tools designed to predict proteins that undergo phase separation. PSPire is a machine learning predictor that integrates sequence and structural features, with particular strength in detecting proteins lacking extensive IDRs. PSPhunter is a machine learning predictor that uses a set of sequence, evolutionary, and functional features. PhaSePred is a meta-predictor that combines multiple established phase separation predictors to provide complementary scores for self-assembling (PS Self) versus partner-dependent (PS Part) phase separation. All three predictors output a score between 0 – 1. Kinases with a p-score >0.7 from all three studies were classified as likely to phase separate (PS), while kinases with a p-score <0.3 from all three studies were classified as not likely to phase separate (No PS). GO term analysis for using pantherdb.org showed that PS kinases were slightly overrepresented (1.4 fold enrichment) with cytosol cellular component but had no significant difference in any other cellular component relative to the entire human kinome.

#### Protein expression and purification

All amino acid sequences of expressed recombinant proteins used for purification are in Table S1.

##### FAK and Abl

His-mEGFP-FAK, His-mEGFP-FAK-W266A, FAK, FAK-W266A, and His-mEGFP-Abl1 were expressed from baculovirus in HighFive^™^ (Thermo) *Trichoplusia ni* cells. Cells were collected by centrifugation and lysed by douncing on ice in 25 mM HEPES (pH 7.5), 30 mM Imidazole (pH 7.5), 500 mM NaCl, 10% glycerol and 5 mM βME + cOmplete(TM), EDTA- free Protease Inhibitor tablet. Centrifugation- cleared lysate was applied to Thermo Scientific^™^ HisPur^™^ Ni-NTA Resin, washed with 25 mM HEPES (pH 7.5), 20 mM Imidazole (pH 7.5), 500 mM NaCl, 10% glycerol, 5 mM βME, 1 μg/ml benzamidine, and eluted with 25 mM HEPES (pH 7.5), 400 mM Imidazole (pH 7.5), 1 M NaCl, 10% glycerol, 5 mM βME, 1 μg/ml benzamidine. Protein was further purified with size exclusion chromatography using a Superdex 200 column (Cytiva) in 25 mM HEPES (pH 7.5), 500 mM NaCl, 10% Glycerol and 1 mM DTT. The His and GFP tags were optionally cleaved at this step using TEV protease treatment for 2 hr at room temperature. Proteins were optionally dephosphorylated at this step by addition of 2x molar equivalents of GST-PTP1B and incubating for 2 hr at room temperature. If TEV treatment and dephosphorylation were both done they were done at the same time. GST-PTP1B was removed at this step by supplementing protein with 20 mM imidazole and applying to Glutathione Sepharose 4B (Cytiva) resin and collecting the flowthrough. TEV treated or dephophorylated protein was further purified with a second round of size exclusion chromatography.

##### GST-PTP1B

BL21(DE3) cells expressing GST- PTP1B were collected by centrifugation and lysed by sonication in 20 mM Tris- HCl (pH 8.0), 200 mM NaCl, 2 mM EDTA (pH 8.0), 1 mM DTT, 1 mM PMSF, 1 μg/ml antipain, 1 μg/ml benzamidine, 1 μg/ml leupeptin, and 1 μg/ml pepstatin. Centrifugation- cleared lysate was applied to Glutathione Sepharose 4B (Cytiva) and washed with 25 mM Tris-HCl (pH 8.0), 200 mM NaCl, and 1 mM DTT. Protein was eluted with 10 mM L-Glutathione (pH 8.0), 50 mM Tris HCL (pH 8.0), 200 mM NaCl, 1mM DTT. Protein was applied to a SOURCE^™^ 15Q anion exchange column and eluted with a gradient of 0 → 1000 mM NaCl in 20 mM HEPES (pH 7.5) and 1 mM DTT. Eluted protein was concentrated using an Amicon Ultra 10 k concentrator and further purified by size exclusion chromatography to remove any protein aggregates using a Superdex 75 prepgrade column (Cytiva) in 50 mM HEPES (pH 7.5), 150 mM NaCl, and 1 mM DTT.

##### IDRs

FAK IDR, Abl IDR, mEGFP-DDX21-CIDR-WT and mEGFP-DDX21-CIDR-R/S proteins were all expressed in Rosetta(DE3) cells with an N-terminal His-SUMO tag. Cells expressing the proteins were collected by centrifugation and lysed by cell disruption (Emulsiflex-C5, Avestin) in 20 mM Tris- HCl (pH 8.0), 300 mM NaCl, 40 mM Imidazole (pH 8.0), 10% glycerol, 1 mM BME, 1 μg/ml antipain, 1 μg/ml benzamidine, 1 μg/ml leupeptin, and 1 μg/ml pepstatin. Centrifugation-cleared lysate was applied to Ni- NTA agarose (Qiagen) and washed with 20 mM Tris- HCl (pH 8.0), 300 mM NaCl, 40 mM Imidazole (pH 8.0), 10% glycerol, 1 mM BME, 1 μg/ml benzamidine and eluted with 20 mM Tris- HCl (pH 8.0), 300 mM NaCl, 300 mM Imidazole (pH 8.0), 10% glycerol, 1 mM BME, 1 μg/ml benzamidine. Protein was applied to a SOURCE^™^ 15Q anion exchange column (Cytiva) and eluted with a gradient of 0 → 1,000 mM NaCl in 20 mM Tris- HCl (pH 8.0), 10% glycerol and 1 mM DTT. Eluted protein was treated with Ulp1 protease to cleave the His-SUMO tag overnight at 4°C. 20 mM imidazole (pH 8.0) was added to the cleaved protein which was then applied to Ni- NTA agarose (Qiagen) and the flowthrough was collected. This was concentrated using Amicon Ultra 10 k concentrators and further purified by size exclusion chromatography to remove any protein aggregates using a Superdex 200 column (Cytiva) in 25 mM HEPES (pH 7.5), 300 mM NaCl, 10% glycerol and 1 mM DTT. For the Abl IDR, the protein was applied to a Source 15S cation exchange column instead of the SOURCE^™^ 15Q and eluted with a gradient of 0→ 1,000 mM NaCl in 25mM HEPES pH 7.5, 10% Glycerol, 1mM BME. The Abl IDR was also cleaved before applying to the SOURCE^™^ 15S cation exchange column.

##### MST2

MST2 protein was expressed in Rosetta(DE3) cells with an N-terminal His-SUMO tag. Cells expressing the protein were collected by centrifugation and lysed by cell disruption (Emulsiflex-C5, Avestin) in 20 mM Tris- HCl (pH 8.0), 300 mM NaCl, 40 mM Imidazole (pH 8.0), 10% glycerol, 1 mM BME, 1 μg/ml antipain, 1 μg/ml benzamidine, 1 μg/ml leupeptin, and 1 μg/ml pepstatin. Centrifugation-cleared lysate was applied to Ni- NTA agarose (Qiagen) and washed with 20 mM Tris- HCl (pH 8.0), 300 mM NaCl, 40 mM Imidazole (pH 8.0), 10% glycerol, 1 mM BME, 1 μg/ml benzamidine and eluted with 20 mM Tris- HCl (pH 8.0), 300 mM NaCl, 300 mM Imidazole (pH 8.0), 10% glycerol, 1 mM BME, 1 μg/ml benzamidine. Protein was applied to a Source 15 Q anion exchange column and eluted with a gradient of 0 → 1,000 mM NaCl in 20 mM Tris- HCl (pH 8.0), 10% glycerol and 1 mM DTT. Eluted protein was treated with Ulp1 protease to cleave the His-SUMO tag overnight at 4°C. 20 mM imidazole (pH 8.0) was added to the cleaved protein which was then applied to Ni- NTA agarose (Qiagen) and the flowthrough was collected. This was concentrated using Amicon Ultra 10 k concentrators and further purified by size exclusion chromatography to remove any protein aggregates using a Superdex 75 column (Cytiva) in 25 mM HEPES (pH 7.5), 300 mM NaCl, and 1 mM DTT.

##### GFP-SAV1-MBP

mEGFP-SAV1-MBP was expressed in Rosetta(DE3) cells. LB media was supplemented with 0.2% glucose. Cells expressing the protein were collected by centrifugation and lysed by cell disruption (Emulsiflex- C5, Avestin) in 20 mM Tris-HCl (pH 7.5), 200 mM NaCl, 1 mM EDTA, 1 mM DTT, 1 μg/ml antipain, 1 μg/ml benzamidine, 1 μg/ml leupeptin, and 1 μg/ml pepstatin. Centrifugation-cleared lysate was applied to Amylose resin (NEB) and washed with 20 mM Tris-HCl (pH 7.5), 200 mM NaCl, 1 mM EDTA, 1 mM DTT, 1 μg/ml benzamidine and eluted with 20 mM Tris-HCl (pH 7.5), 200 mM NaCl, 10 mM maltose, 1 mM EDTA, 1 mM DTT, 1 μg/ml benzamidine. This was concentrated using Amicon Ultra 10 k concentrators and further purified by size exclusion chromatography to remove any protein aggregates using a Superdex 200 column (Cytiva) in 25 mM HEPES (pH 7.5), 300 mM NaCl, and 1 mM DTT.

##### P130Cas

His_6_-p130Cas was expressed from baculovirus in Spodoptera frugiperta (Sf9) cells. Cells were collected by centrifugation and lysed by douncing on ice in 20 mM Tris-HCl (pH 8.0), 20 mM Imidazole (pH 8.0), 500 mM NaCl, 10% glycerol and 5 mM βME + cOmplete, EDTA-free Protease Inhibitor tablet (Roche). Centrifugation-cleared lysate was applied to Ni-NTA agarose beads (Qiagen), washed with 20 mM Tris-HCl (pH 8.0), 20 mM Imidazole (pH 8.0), 500 mM NaCl, 10% glycerol and 5 mM βME, and then eluted with 20 mM Tris-HCl (pH 8.0), 400 mM Imidazole (pH 8.0), 500 mM NaCl, 10% glycerol and 5 mM βME. The His tag was removed using TEV protease treatment for 16 hr at 4 °C. Cleaved protein was applied to a Source 15 Q anion exchange column and eluted with a gradient of 100 → 300 mM NaCl in 20 mM Immidazole (pH 7.0), 1 mM DTT and 10% glycerol. Collected fractions were concentrated (Amicon 50 K, Millipore) and applied to an SD200 column in 25 mM Hepes pH7.5, 150 mM NaCl and 10% glycerol. Cas was concentrated using Amicon Ultra Centrifugal Filter units (Millipore) to >400 μ M, mixed with 100 mM HEPES (pH 7.5), 100 mM NaCl, 15 mM ATP, 20 mM MgCl_2_, 2 mM DTT, and 150 nM active Lck, and incubated for 16 hrs at 30°C. Fully phosphorylated Cas was purified by size exclusion chromatography to remove any protein aggregates using a Superdex 200 prepgrade column (GE Healthcare) in 25 mM HEPES (pH 7.5), 150 mM NaCl, 1 mM βME, and 10% glycerol. Cas phosphorylation was confirmed by size shift in SDS-PAGE gel and quantified with mass spectrometry.

##### Paxillin

BL21(DE3) cells expressing GST-Paxillin were collected by centrifugation and lysed by cell disruption (Emulsiflex-C5, Avestin) in 20 mM Tris-HCl (pH 8.0), 300 mM NaCl, 0.01% NP-40, 10% glycerol, 1 mM DTT, 1 μg/ml antipain, 1 μg/ml benzamidine, 1 μg/ml leupeptin, and 1 μg/ml pepstatin. Centrifugation-cleared lysate was applied to Glutathione Sepharose 4B (GE Healthcare) and washed with 20 mM Tris-HCl (pH 8.0), 300 mM NaCl, 0.01% NP-40, 10% glycerol, 1 mM DTT, 1 μg/ml benzamidine. GST was cleaved from protein by TEV protease treatment for 16 hr at 4°C. Cleaved protein was applied to a Source 15 Q anion exchange column and eluted with a gradient of 5 → 500 mM NaCl in 20 mM imidazole (pH 8.0), 10% glycerol and 1 mM DTT. Eluted protein was concentrated using Amicon Ultra 10 k concentrators and further purified by size exclusion chromatography to remove any protein aggregates using a Superdex 200 column (GE Healthcare) in 25 mM HEPES (pH 7.5), 150 mM NaCl, and 1 mM βME.

##### Nck

BL21(DE3) cells expressing GST-Nck were collected by centrifugation and lysed by sonication in 25 mM Tris-HCl (pH 8.0), 200 mM NaCl, 2 mM EDTA (pH 8.0), 1 mM DTT, 1 mM PMSF, 1 μg/ml antipain, 1 μg/ml benzamidine, 1 μg/ml leupeptin, and 1 μg/ml pepstatin. Centrifugation-cleared lysate was applied to Glutathione Sepharose 4B (GE Healthcare) and washed with 20 mM Tris-HCl (pH 8.0), 200 mM NaCl, and 1 mM DTT. GST was cleaved from protein by TEV protease treatment for 16 hr at 4°C. Cleaved protein was applied to a Source 15 Q anion exchange column and eluted with a gradient of 5 → 250 mM NaCl in 20 mM imidazole (pH 7.0) and 1 mM DTT. Eluted protein was pooled and applied to a Source 15 S cation exchange column and eluted with a gradient of 0 → 500 mM NaCl in 20 mM imidazole (pH 7.0) and 1 mM DTT. Eluted protein was concentrated using Amicon Ultra 10 k concentrators and further purified by size exclusion chromatography to remove any protein aggregates using a Superdex 75 prepgrade column (GE Healthcare) in 25 mM HEPES (pH 7.5), 150 mM NaCl, and 1 mM βME.

##### N-WASP

BL21(DE3) cells expressing His_6_-N-WASP were collected by centrifugation and lysed by cell disruption (Emulsiflex-C5, Avestin) in 20 mM imidazole (pH 7.0), 300 mM KCl, 5 mM βME, 0.01% NP-40, 1 mM PMSF, 1 μg/ml antipain, 1 μg/ml benzamidine, 1 μg/ml leupeptin, and 1 μg/ml pepstatin. The cleared lysate was applied to Ni-NTA agarose (Qiagen), washed with 50 mM imidazole (pH 7.0), 300 mM KCl, 5 mM βME, and eluted with 300 mM imidazole (pH 7.0), 100 mM KCl, and 5 mM βME. The elute was further purified over a Source 15 Q column using a gradient of 250 → 450 mM NaCl in 20 mM imidazole (pH 7.0), and 1 mM DTT. The His_6_-tag was removed by TEV protease at 4°C for 16 hr (for His-N-WASP, no TEV treatment occurred). Cleaved N-WASP (or uncleaved for His-N-WASP) was then applied to a Source 15 S column using a gradient of 110 → 410 mM NaCl in 20 mM imidazole (pH 7.0), 1 mM DTT. Fractions containing N-WASP were concentrated using an Amicon Ultra 10 k concentrator (Millipore) and further purified by size exclusion chromatography to remove any protein aggregates using a Superdex 200 prepgrade column (GE Healthcare) in 25 mM HEPES (pH 7.5), 150 mM KCl, 1 mM βME, and 10% glycerol.

##### TEV protease

His6-TEV protease protein was expressed in Rosetta(DE3) cells with an N-terminal His-SUMO tag. Cells expressing the protein were collected by centrifugation and lysed by cell disruption (Emulsiflex- C5, Avestin) in 20mM Tris HCl pH 8.0, 25mM Imidazole pH 8.0, 200mM NaCl, 10% glycerol, 5mM BME, 1 mM PMSF. Centrifugation-cleared lysate was applied to Ni- NTA agarose (Qiagen) and washed with 20 mM Tris- HCl (pH 8.0), 200 mM NaCl, 25 mM Imidazole (pH 8.0), 10% glycerol, 5 mM BME and eluted with 20 mM Tris- HCl (pH 8.0), 200 mM NaCl, 300 mM Imidazole (pH 8.0), 10% glycerol, 5 mM BME. Protein was applied to a Source 15 S cation exchange column and eluted with a gradient of 0 → 1,000 mM NaCl in 20 mM HEPES (pH 7.0), 10% glycerol and 2 mM DTT. Eluted protein was concentrated using Amicon Ultra 10k concentrators and further purified by size exclusion chromatography to remove any protein aggregates using a Superdex 75 column (Cytiva) in 20 mM Tris (pH 8.0), 150 mM NaCl, 10% Glycerol and 2 mM DTT.

##### Ulp1 protease

His6-Ulp1 protease protein was expressed in BL21 (DE3) cells with an N-terminal His-SUMO tag. Cells expressing the protein were collected by centrifugation and lysed by cell disruption (Emulsiflex- C5, Avestin) in 50 mM NaH_2_PO_4_ pH 8.0, 10 mM Imidazole pH 8.0, 300mM NaCl, 0.1% IGEPAL, 5mM BME. Centrifugation-cleared lysate was applied to Ni- NTA agarose (Qiagen) and washed with 50 mM NaH_2_PO_4_ pH 8.0, 20 mM Imidazole pH 8.0, 300mM NaCl, 5mM BME and eluted with 50 mM NaH_2_PO_4_ pH 8.0, 250 mM Imidazole pH 8.0, 300mM NaCl, 5mM BME. Protein was applied to a Source 15 Q anion exchange column and eluted with a gradient of 0 → 1,000 mM NaCl in 20 mM Tris-HCl (pH 8.0), 10% glycerol and 1 mM DTT. Eluted protein was concentrated using Amicon Ultra 10k concentrators and further purified by size exclusion chromatography to remove any protein aggregates using a Superdex 75 column (Cytiva) in 25 mM HEPES (pH 7.5), 300 mM NaCl, 10% Glycerol and 1 mM DTT.

##### Lck

His6-Lck was expressed from baculovirus in Spodoptera frugiperta (Sf9) cells. Cells were collected by centrifugation and lysed by douncing on ice in 50 mM Tris-HCl (pH 7.5), 100 mM NaCl, 5 mM βME, 0.01% NP-40, 1 mM PMSF, 1 μg/ml antipain, 1 μg/ml benzamidine, and 1 μg/ml leupeptin. Centrifugation-cleared lysate was applied to Ni-NTA agarose beads (Qiagen), washed with 20 mM Tris-HCl (pH 7.5), 1 M NaCl, 20 mM imidazole (pH 7.5), 5 mM βME, and 10% glycerol, and then eluted with 20 mM Tris-HCl (pH 7.5), 100 mM NaCl, 200 mM imidazole (pH 7.5), 5 mM βME, and 10% glycerol. The eluate was applied to a Source 15 Q anion exchange column and eluted with a gradient of 100 → 300 mM NaCl in 25 mM HEPES (pH 7.5) and 2 mM βME. Collected fractions were concentrated (Amicon 10K, Millipore) and applied to an SD75 column in 25 mM HEPES (pH 7.5), 150 mM NaCl, and 1 mM βME.

#### Fluorophore conjugation

For conjugation with maleimide chemistry, recombinant proteins to be labeled with Alexa fluorophores were concentrated using Amicon Ultra Centrifugal Filter units (Millipore) to ~100 μM. 5 mM βME was added to reduce cysteine residues followed by buffer exchange using a HiTrap 26/10 Desalting column (Cytiva) in protein storage buffer (see protein purification protocol) without any reducing agent. Fractions containing protein were collected and concentrated to 100 μM. 500 μM Alexa Fluor 647C2 Maleimide (ThermoFisher) was added, and the reaction was incubated at 4°C for 16 hr. The reaction was quenched with 1 μl 14.3 M βME followed by final buffer exchange using a HiTrap 26/10 Desalting column (Cytiva) in protein storage buffer (see protein purification protocol) with reducing agent. Final protein concentration and degree of labeling were calculated from the protein absorbance using the following formula. “ExtCoef” is the extinction coefficient of the protein being labeled.


$$\;protein\;concentration\;(M)=\frac{A_{280}-(A_{650}\times 0.03)}{ExtCoef}\;Degree\;of\;labeling=\frac{A_{650}}{239,000\times [protein\;concentration\;(M)]}$$

#### Visualization

The PhotoMol webtool was used to graph normalized counts of mass photometry data for each sample.^60^

Diagrams of FAK, Abl and DDX21 domains and IDRs were made with IBS 2.0 webtool.^61^

Dendrogram of human kinome was made with KinMap.^62^

Diagrams of experimental setups (Figures 1A, 1F, 2A, and 4B) were created with BioRender.com.

Figure 1A: Created in BioRender. Case, L. (2026) https://BioRender.com/ydwi48j.

Figure 1F: Created in BioRender. Case, L. (2026) https://BioRender.com/o5753hc.

Figure 2A: Created in BioRender. Case, L. (2026) https://BioRender.com/phz7td7.

Figure 4B: Created in BioRender. Case, L. (2026) https://BioRender.com/9cfcndp.

### QUANTIFICATION AND STATISTICAL ANALYSIS

Statistical analysis was performed in GraphPad Prism. All statistical details of experiments can be found in the figure legends.

## Supplementary Material

1

2

3

Supplemental information can be found online at https://doi.org/10.1016/j.celrep.2026.117459.

## Figures and Tables

**Table:** KEY RESOURCES TABLE

REAGENT or RESOURCE	SOURCE	IDENTIFIER
Antibodies		
Mouse Anti-FAK, clone 4.47	Millipore Sigma	Cat#05-537; RRID: AB_2173817
Invitrogen^™^ Phospho-FAK (Tyr397) Rabbit Polyclonal Antibody	Fisher Scientific	Cat#44-624-G; RRID: AB_2533701
Goat Anti-mouse IgG (H+L) (DyLight^®^ 680 Conjugate) #5470	Cell Signaling Technology	Cat#5470; RRID: AB_10696895
Goat Anti-rabbit IgG (H+L) (DyLight^®^ 800 4X PEG Conjugate) #5151	Cell Signaling Technology	Cat#5151; RRID: AB_10697505
BD Transduction Laboratories^™^ Purified Mouse Anti-p130 [Cas]	BD Biosciences	Cat#610271; RRID: AB_397666
Rabbit anti pY410 p130 Cas	Cell Signaling Technology	Cat#4011; RRID: AB_2274823
BD Transduction Laboratories^™^ Purified Mouse Anti-Paxillin	BD Biosciences	Cat#612405; RRID: AB_647289
Rabbit anti pY118 paxillin #2451	Cell Signaling Technology	Cat#2541; RRID: AB_2174466
Rabbit anti EEA1#2411	Cell Signaling Technology	Cat#2411; RRID: AB_2096814
Rab11 (D4F5) Rabbit Monoclonal Antibody #5589	Cell Signaling Technology	Cat#5589; RRID: AB_10693925
Donkey anti-Rabbit IgG (H+L) Highly Cross-Adsorbed Secondary Antibody, Alexa Fluor^™^ Plus 647	Fisher Scientific	Cat#PIA32795TR; RRID: AB_2762835
Phospho-MST1 (Thr183)/MST2 (Thr180) (E7U1D) Rabbit Monoclonal Antibody #49332	Cell Signaling Technology	Cat#49332; RRID: AB_2799355
STK3 Mouse Monoclonal Antibody (4F7)	Thermo Fisher Scientific	Cat#H00006788-M13A; RRID: AB_1710171
Phospho-c-Abl (Tyr245) Rabbit Antibody #2861	Cell Signaling Technology	Cat#2861; RRID: AB_331029
c-Abl Mouse Antibody (24-11)	Santa Cruz Biotechnology	Cat#sc-23; RRID: AB_626775
Bacterial and virus strains		
BL21(DE3) Competent *E*. *coli*	New England Biolabs	Cat#C2527H
MilliporeSigma^™^ Novagen^™^ Rosetta^™^ 2 (DE3) Singles Competent Cells	Fisher Scientific	Cat#71-400-3
DH10αEMBacY *E*. *coli*	Vos laboratory	N/A
NEB^®^ 5-alpha Competent *E*. *coli*	New England Biolabs	Cat#C2987H
Chemicals, peptides, and recombinant proteins		
Hellmanex^™^ III	Millipore Sigma	Cat#Z805939
Creative Pegworks MPEG-SILANE MW 5K 1G	Fisher Scientific	Cat#NC1919934
Poly-D-Lysine	Thermo Fisher Scientific	Cat#A3890401
Bovine Fibronectin	Millipore Sigma	Cat#F1141
Doxycycline hyclate	Millipore Sigma	Cat#D5207
RIPA Lysis and Extraction Buffer	Thermo Fisher Scientific	Cat#89900
Pierce^™^ Bovine Serum Albumin Standard, 2 mg/mL	Thermo Fisher Scientific	Cat#23210
Fetal Bovine Serum, Tet system approved, USDA-approved regions	Thermo Fisher Scientific	Cat#A4736401
AzureRed Fluorescent Total Protein Stain	Azure Biosystems	Cat#AC2124
Azure Fluorescent Blot Blocking Buffer	Azure Biosystems	Cat#2190
Azure Fluorescent Blot Washing Buffer	Azure Biosystems	Cat#2145
Glucose oxidase	Millipore Sigma	Cat#G2133
Catalase	Millipore Sigma	Cat#C1345
FluoSpheres^™^ Carboxylate-Modified Microspheres	ThermoFisher Scientific	Cat#F8812
NativeMark^™^ Unstained Protein Standard	ThermoFisher Scientific	Cat#LC0725
Invitrogen^™^ Lipofectamine^™^ 3000 Transfection Reagent	Fisher Scientific	Cat#L3000008
Halt^™^ Protease and Phosphatase Inhibitor Cocktail	ThermoFisher Scientific	Cat#78440
Paraformaldehyde	Fisher Scientific	Cat#AA433689M
Triton^™^ X-100	Millipore Sigma	Cat#X100
TWEEN^®^ 20	Millipore Sigma	Cat#P9416
Saponin	Millipore Sigma	Cat#47036
Benzamidine	Millipore Sigma	Cat#B6506
Antipain	Millipore Sigma	Cat#A6191
Leupeptin	Millipore Sigma	Cat#L2884
Pepstatin	Millipore Sigma	Cat#P4265
L-Glutathione	Millipore Sigma	Cat#G4251
Source 15 Q Anion Exchange Resin	Cytiva	Cat#17094705
Source 15 S Cation Exchange Resin	Cytiva	Cat#17094401
Amylose resin	New England Biolabs	Cat#E8021L
X-tremeGENE^™^ 9 DNA Transfection Reagent	Millipore Sigma	Cat#6365779001
Critical commercial assays		
Invitrogen^™^ ATP Determination Kit	Fisher Scientific	Cat#A22066
ADP-Glo^™^ Kinase Assay	Promega	Cat#V6930
GTPase-Glo^™^ Assay	Promega	Cat#V7681
Micro BCA^™^ Protein Assay Kit	ThermoFisher Scientific	Cat#23235
Q5^®^ Site-Directed Mutagenesis Kit	New England Biolabs	Cat#E0554S
Deposited data		
Phospho-MS Data	This study	Dryad: https://doi.org/10.5061/dryad.j3tx95xwc
Experimental models: Cell lines		
Mouse: MEFs: FAK +/+	ATCC	Cat#CRL-2645
Insect: Sf9 Cells	Vos laboratory	N/A
Insect: Sf21 Cells	Vos laboratory	N/A
Insect: HighFive^™^ Trichoplusia ni Cells	Vos laboratory	N/A
Recombinant DNA		
XLone-Puro-EGFP	Addgene	Cat#140027
XLone-Puro-mEGFP-FAK-WT	This study	N/A
Super PiggyBac Transposase	Calo laboratory	N/A
pFAST-His-TEV-mEGFP-TEV-FAK-WT	This study	N/A
pFAST-His-TEV-mEGFP-TEV-FAK-W266A	This study	N/A
pCMV-mEGFP-FAK-WT	Addgene	Cat#186148
pCMV-mEGFP-FAK-W266A	Addgene	Cat#186149
pCMV-mCherry-Paxillin	This study	N/A
pFAST-His-mEGFP-TEV-Abl-WT	This study	N/A
pFAST-His-mEGFP-TEV-Abl-RS	This study	N/A
pCIOX-Mst2	This study	N/A
pCIOX-mEGFP-Sav1-MBP	This study	N/A
pCIOX-FAK-IDR	This study	N/A
pCIOX-Abl-IDR	This study	N/A
pCIOX-DDX21-C-IDR-WT	This study	N/A
pCIOX-DDX21-C-IDR-RS	This study	N/A
pGEX-PTP1B	This study	N/A
pFAST-His-p130Cas	Addgene	Cat#186131
pGEX-Paxillin	Addgene	Cat#186142
pGEX-Nck	Addgene	Cat#186133
pHis-N-WASP	Addgene	Cat#186132
His-Ulp1Protease	Addgene	Cat#64697
His-MBP-TEV Protease	Addgene	Cat#92414
Software and algorithms		
ImageJ	Schindelin et al.	
LasX Acquisition	Leica Microsystems, GmbH	
Refeyn Discover MP	Refeyn, Ltd	
Metamorph Acquisition	Molecular Devices, LLC	
GraphPad Prism	GraphPad Software, LLC	
Other		
Superdex 200 Size Exclusion Column	Cytiva	Cat#28989335
Superdex 75 Size Exclusion Column	Cytiva	Cat#28989333
HiTrap 26/10 Desalting column	Cytiva	Cat#17140801

## Highlights

- Phase separation concentrates kinases to trigger *trans*-autophosphorylation
- Kinase condensates enrich ATP through non-stereospecific, electrostatic interactions
- Positively charged IDRs drive ATP enrichment in condensates
