# Supplementary material for: Kinase condensates enrich ATP and trigger autophosphorylation
Source: Cell Rep. Author manuscript; Available in PMC 2026 Aug 3. (PMC13430976; doi:10.1016/j.celrep.2026.117459)
Supplement: 1 [file NIHMS2190735-supplement-1.pdf]

Cell Reports, Volume *45*

Supplemental information

Kinase condensates enrich ATP and trigger autophosphorylation

Nicholas E. Lea and Lindsay B. Case

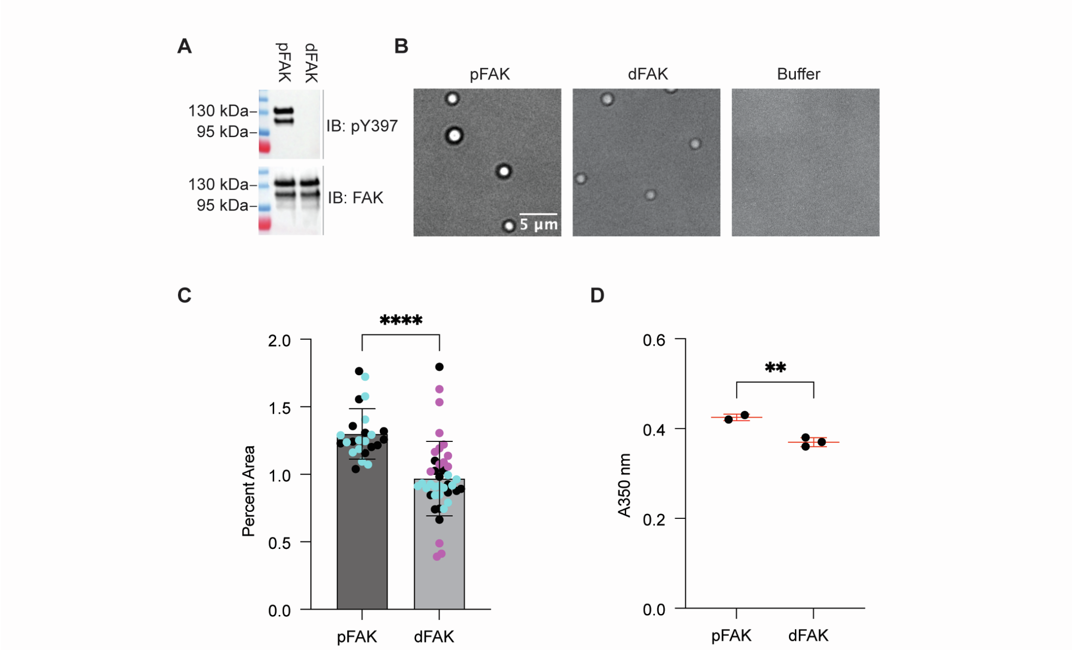

Fig. S1. Tyrosine phosphorylation is not required for FAK phase separation, related to Figure 1. (**A**) Chemiluminescent western blots against total and phospho-Y397 FAK of tyrosine dephosphorylated (dFAK) and native phosphorylated FAK (pFAK). (**B**) DIC microscopy images of pFAK and dFAK condensates at 1 μM and buffer only control. Scale bar is 5 microns. (**C**) Quantification of percent droplet area for data in (B). Each point represents a single field of view. N=24 for pFAK and N=41 for dFAK. Colors correspond to field of views from the same well. (**D**) Turbidity measurements of pFAK and dFAK at 2 μM. N=2 for pFAK and N=3 for dFAK. For all graphs error bars are standard deviation and significance was tested with unpaired T-tests. (* p<0.0332, ** p<0.0021, *** p<0.0002, **** p<0.0001). For all experiments the buffer was 25 mM HEPES pH 7.5, 50 mM NaCl, 1% glycerol, 1 mM DTT.

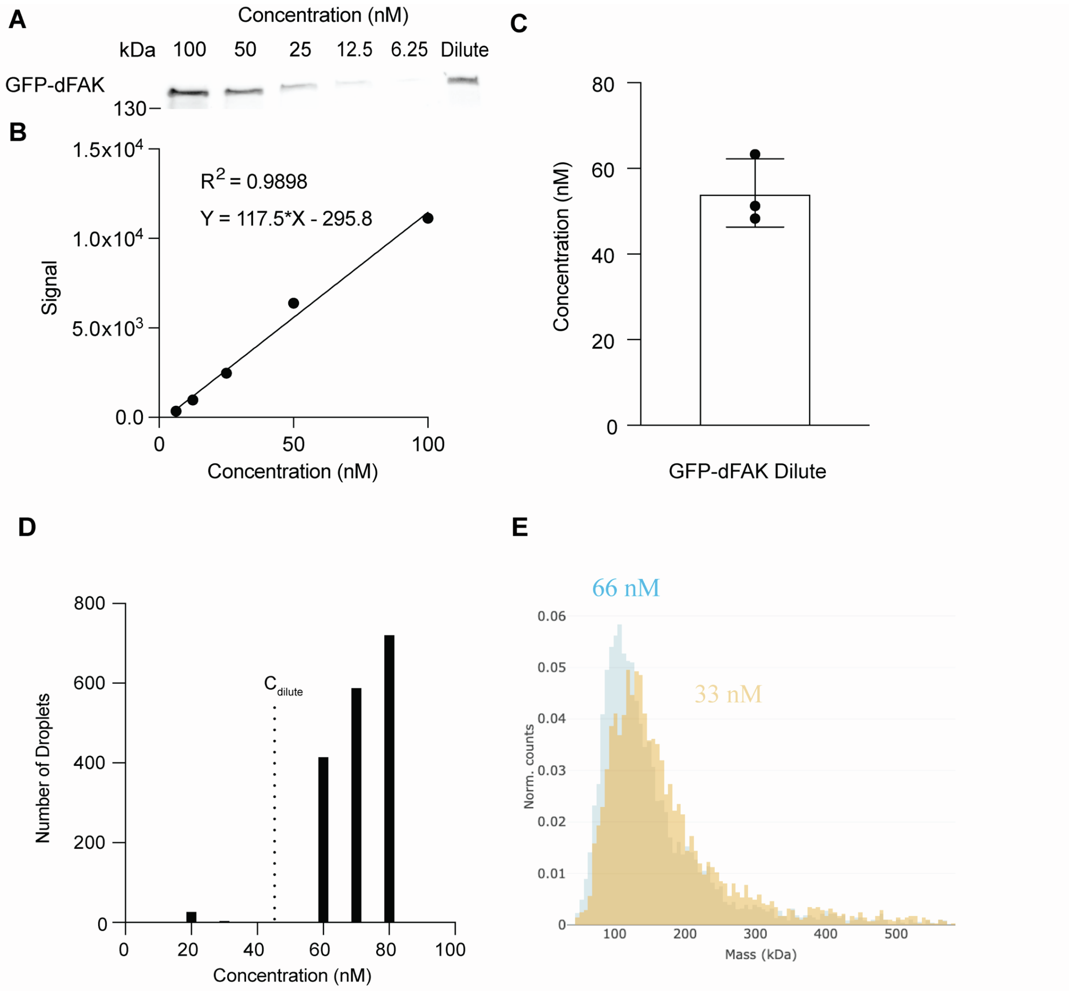

Fig. S2. FAK condensates form above the measured C_{dilute} in vitro with no detectable change in dilute phase oligomerization, related to Figure 1. (**A**) Representative quantitative western blot of mEGFP-dFAK standards and dilute phase sample after sedimentation. (**B**) Quantification of bands in (A) and associated linear regression. (**C**) Quantification of mEGFP-dFAK dilute phase concentration measured from quantitative western blots (N=3 replicates). Error bars are standard deviation. (**D**) Quantification of number of droplets across 10 fields of view for 10, 20, 30, 60, 70, and 80 nM GFP-dFAK. (**E**) Mass photometry measurements of mEGFP-FAK protein in dilute phase below (orange; 33 nM) and above (blue; 66 nM) the C_{dilute} concentration. For all experiments the buffer was 25 mM HEPES pH 7.5, 50 mM NaCl, 1% glycerol, 1 mM DTT.

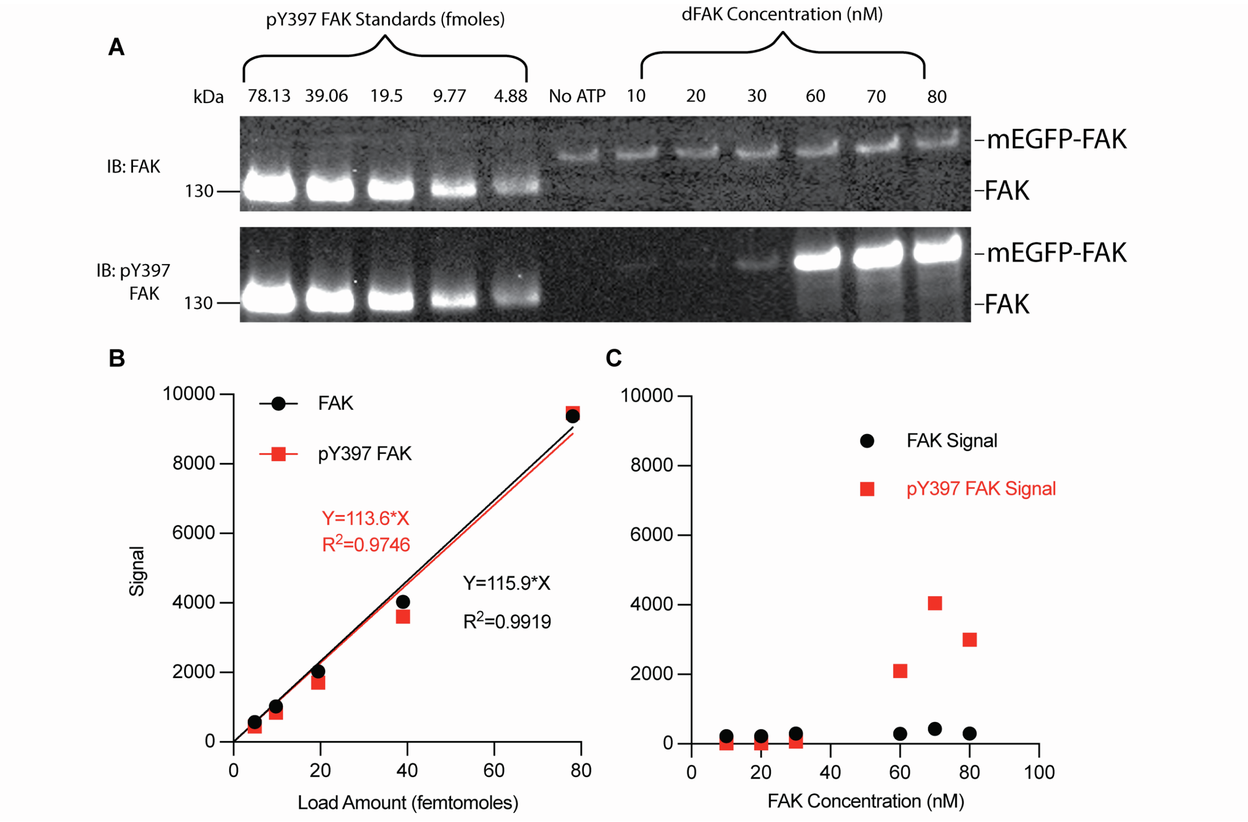

Fig. S3. Western blots accurately measure FAK autophosphorylation, related to Figure 1. (**A**) Images of multichannel fluorescent western blots of pY397 FAK standards run on the same blot as samples from experiment in Fig 1L-M. FAK signal is from an anti-mouse DyLight® 680 secondary antibody and pY397 FAK signal is from an anti-rabbit DyLight® 800 secondary antibody. (**B**) Linear regressions of FAK and pY397 FAK signals from blot in (A). (**C**) FAK and pFAK signals from samples in (A). To obtain "total activity", the pY397 signals were divided by the FAK signals to correct for loading differences. Since all samples were diluted to the same 10 nM concentration before loading on SDS-PAGE, these normalized pY397 values were further multiplied by their respective dilution factors to yield the "activity" or amount of pY397 FAK in each sample. These dilution factors were 2.75, 5.5, 8.25, 16.5, 19.25, and 22 for 10, 20, 30, 60, 70 and 80 nM respectively. The 60 nM sample has a ~25-fold increase in activity compared to the 30 nM sample. For some samples below 60 nM the pY397 signal was significantly below the pY397 signal of the lowest standard. To make a conservative estimate of the activity increase between 30 and 60 nM, we can assume that the pY397 signal is equal to that of the lowest loaded standard. If we do so, the 60 nM sample has a ~10 fold increase in activity compared to the 30 nM sample.

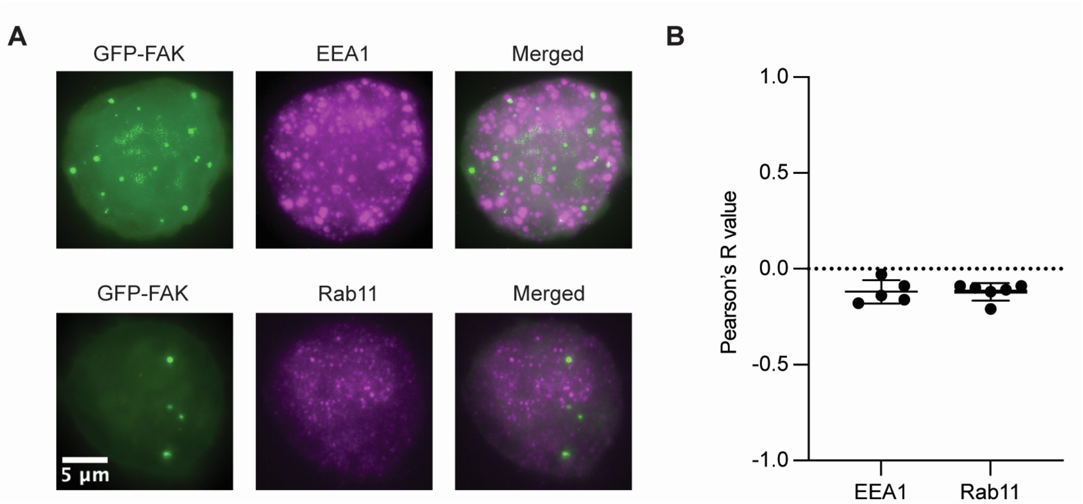

Fig S4. mEGFP-FAK cytoplasmic puncta do not co-localize with early or recycling endosomes, related to Figure 2. (**A**) Immunostaining of early (EEA1) and recycling (Rab11) endosomal markers in mEGFP-FAK-WT expressing cells plated on poly-D-Lysine. Scale bar is 5 microns. (**B**) Colocalization analysis of data in (A). Colocalization analysis was performed on mEGFP-FAK puncta and EEA1 and Rab11 endosomes for N=5 and N=6 immunostained cells, respectively. Analysis was limited to cells containing mEGFP-FAK puncta. Error bars represent standard deviation.

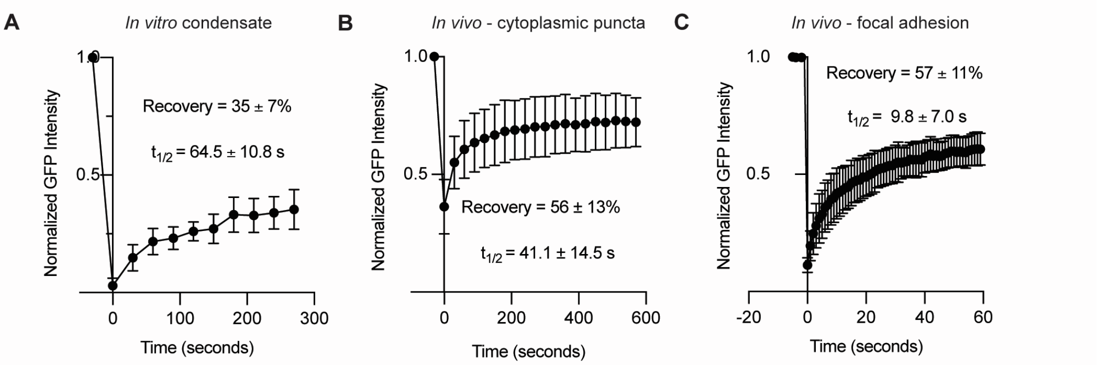

Fig S5. mEGFP-FAK displays faster and higher FRAP recovery in cytoplasmic puncta and focal adhesions compared to in vitro condensates, related to Figure 2. (**A**) FRAP data of mEGFP-FAK condensates in vitro. Buffer was 25 mM HEPES pH 7.5, 50 mM NaCl, 1% glycerol, 1 mM DTT (N = 4 replicates). (**B**) FRAP data of cytoplasmic mEGFP-FAK puncta (**I**dentical data from Fig. 2C placed here for reference). All measurements were taken within 30 minutes of plating on poly-D-Lysine (N=5 replicates). (**C**) FRAP data of mEGFP-FAK at focal adhesions. All measurements were taken 2 hours after plating on fibronectin (N=6 replicates). For all plots, error bars represent standard deviation and error of recovery and $t_{1/2}$ are mean and standard deviations.

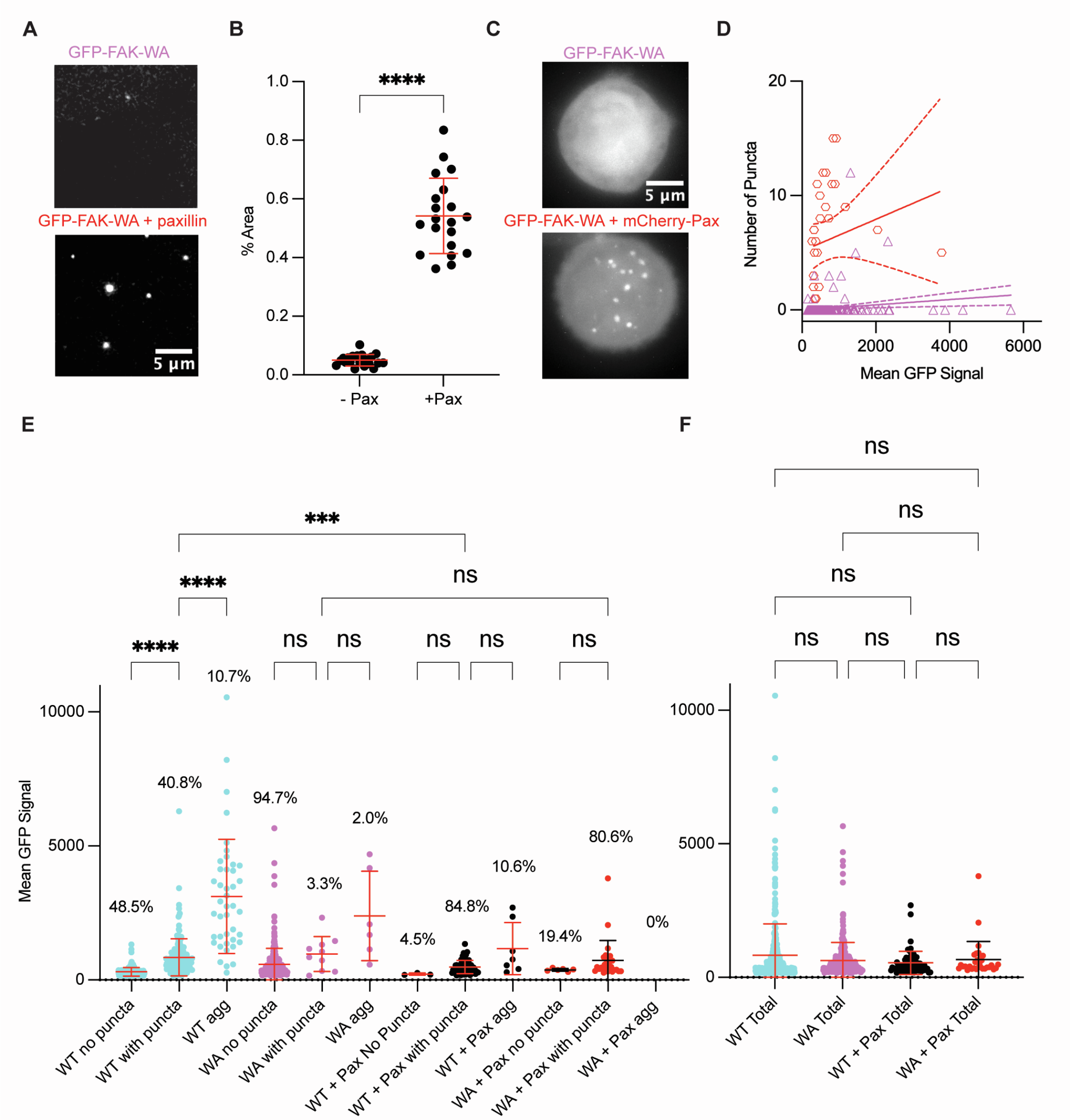

Fig. S6. mEGFP-FAK puncta phenotypes in cells recapitulates in vitro treatments, related to Figure 2. (**A**) Representative images of mEGFP-FAK-WA and mEGFP-FAK-WA + paxillin condensates in vitro. Final concentration of mEGFP-FAK and paxillin was 0.5 µM. Buffer: 25 mM HEPES pH 7.5, 50 mM NaCl, 1% glycerol, 1 mM DTT. Scale bar is 5 microns. (**B**) Quantification of percent area of condensates for both conditions in (A). Data is from 2 replicates (N=20 images). Significance was tested with unpaired t-test with Welch's correction. (**C**) Representative maximum intensity projections of MEFs plated on poly-D-Lysine. Cells chosen have a GFP signal of ~1000 a.u. Scale bar is 5 microns. (**D**) Quantification of number of puncta vs. total GFP signal. For cells co-expressed with paxillin, analyzed cells had an mCherry-Pax signal between 300-600 a.u. Dotted lines represent 95% confidence intervals of linear regressions. (**E**) Mean GFP signal of cells binned by puncta phenotypes (ie. no puncta, puncta, or aggregates). Percentages are the percent of transfected cells with each phenotype for each condition. (**F**) Mean GFP signal of all transfected cells for each condition. For (E) and (F) significance was tested by Kruskal-Wallis test followed by a Dunn's multiple comparison test. For all graphs error bars represent standard deviation. * $p<0.0332$, ** $p<0.0021$, *** $p<0.0002$, **** $p<0.0001$.

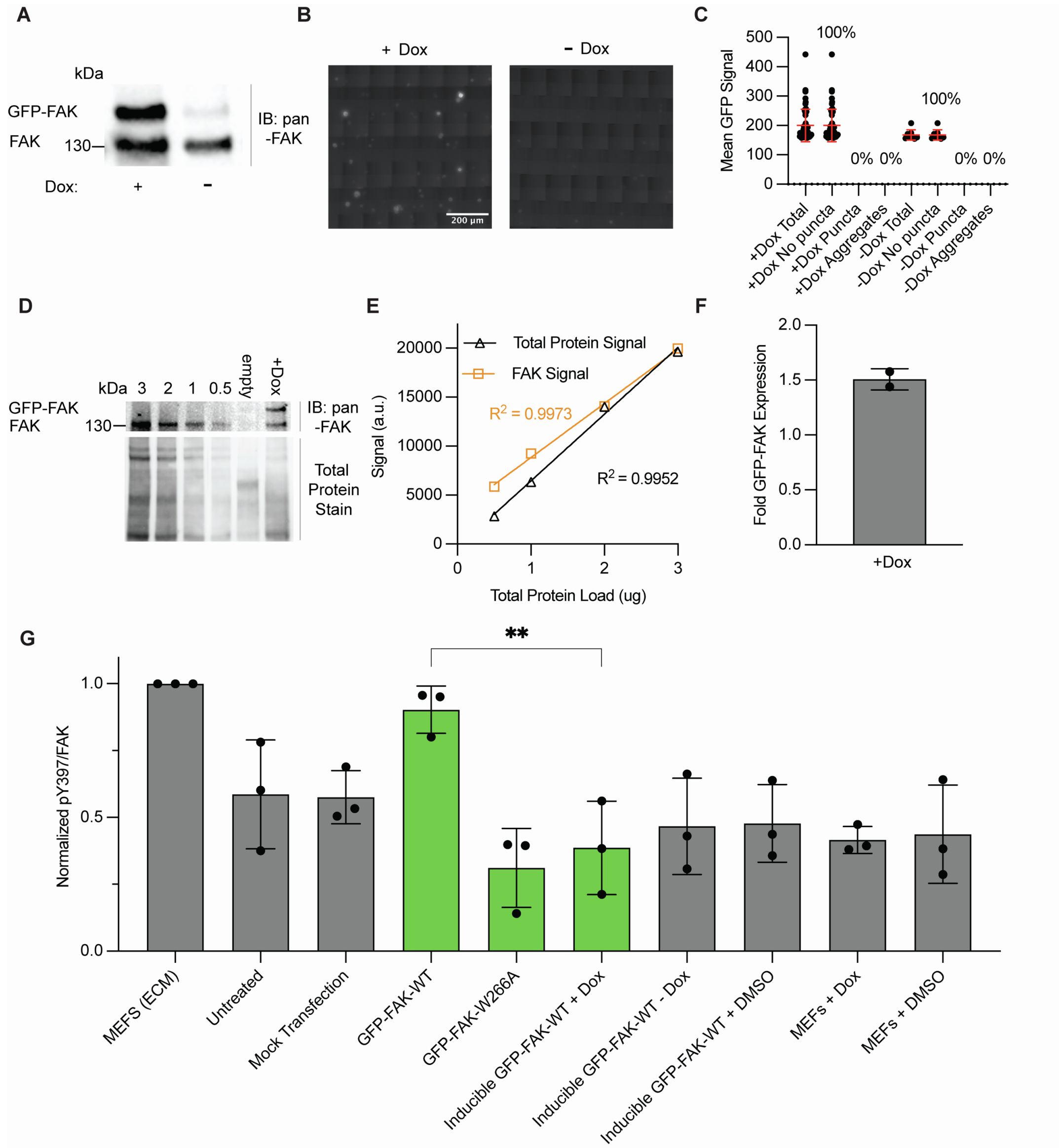

Fig. S7. mEGFP-FAK overexpression is not sufficient to rescue integrin-dependent pY397/FAK ratios when plated on poly-D-Lysine, related to Figure 2. (**A**) Representative western blot of stable Dox-inducible mEGFP-FAK MEF lysates with and without doxycycline treatment. (**B**) Images of stable Dox-inducible mEGFP-FAK MEF cells plated on poly-D-Lysine with and without doxycycline treatment. Images are stitched together. Scale bar is 200 microns. (**C**) Mean GFP signal for total populations of transfected MEFs or binned by puncta phenotypes (i.e.. no puncta, puncta, or aggregates). No cells exhibited puncta indicating that the overexpression levels in these experiments are below the concentration required for mEGFP-FAK phase separation. (**D**) Western blot and total protein stain of cell lysates from untransfected MEFs or Dox-inducible mEGFGP-FAK MEFs incubated with doxycycline for 24 hours. (**E**) Linearity of FAK channel and total protein stain was confirmed with dilutions of lysates. (**F**) Quantification of fold mEGFP-FAK expression from western blot in (D) for N=2 replicates. FAK was normalized to total protein stain and values represent fold expression compared to MEFs. (**G**) pY397/FAK ratios determined by western blot analysis of cell lysates. All cells were plated on poly-D-Lysine for 30 minutes before lysis except MEFS (ECM), which were grown on culture treated dishes for 24 hours. Green bars indicate analysis performed on mEGFP-FAK and grey bars on endogenous FAK. Significance tested by one-way ANOVA followed by a Tukey multiple comparison test (* $p<0.0332$, ** $p<0.0021$, *** $p<0.0002$, **** $p<0.0001$). For all graphs error bars represent standard deviation.

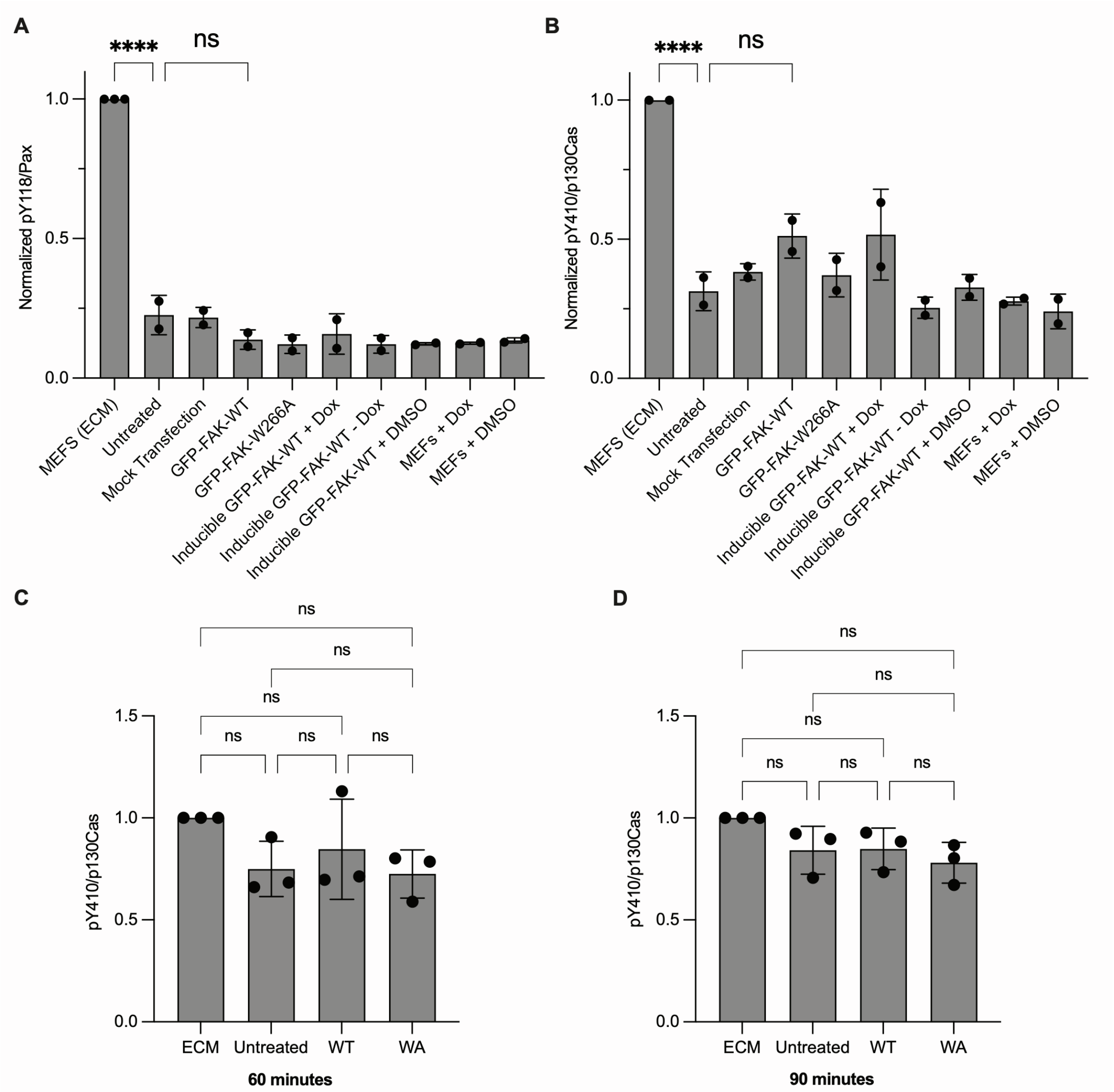

Fig. S8. Cytoplasmic FAK condensates are not sufficient to rescue paxillin or p130Cas phosphorylation independent of integrin-based adhesion, related to Figure 2. (**A**) pY118 Pax/Pax ratios determined by western blot analysis of MEF lysates. N=2 replicates. (**B**) pY410 Cas/p130Cas ratios determined by western blot analysis of MEF lysates. N=2 replicates. All cells were plated on poly-D-Lysine for 30 minutes before lysis except MEFS (ECM), which were grown on culture treated dishes for 24 hours. (**C**) pY410 Cas/p130Cas ratios determined by western blot analysis of MEF lysates. N=3 replicates. All cells were plated on poly-D-Lysine for 60 minutes before lysis. (**D**) pY410 Cas/p130Cas ratios determined by western blot analysis of MEF lysates. N=3 replicates. All cells were plated on poly-D-Lysine for 90 minutes before lysis. Significance tested by one-way ANOVA followed by a Tukey multiple comparison test (* p<0.0332, ** p<0.0021, *** p<0.0002, **** p<0.0001). For all graphs error bars represent standard deviation.

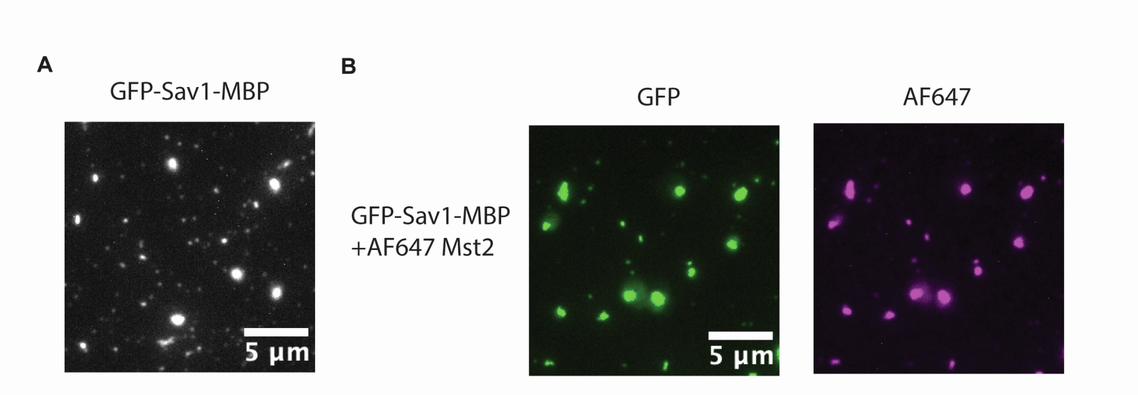

Fig. S9. mEGFP-SAV1-MBP forms condensates that enrich Mst2 in vitro, related to Figure 3. (**A**) Image of GFP-Sav1-MBP at 100 nM concentration. Final buffer composition is 25 mM HEPES pH 7.5, 100 mM NaCl, 10% PEG8000, 1 mM DTT. (**B**) Images of GFP-Sav1-MBP and AF647-Mst2 at 1 µM concentration. Final buffer composition is identical to (A). All scale bars are 5 microns.

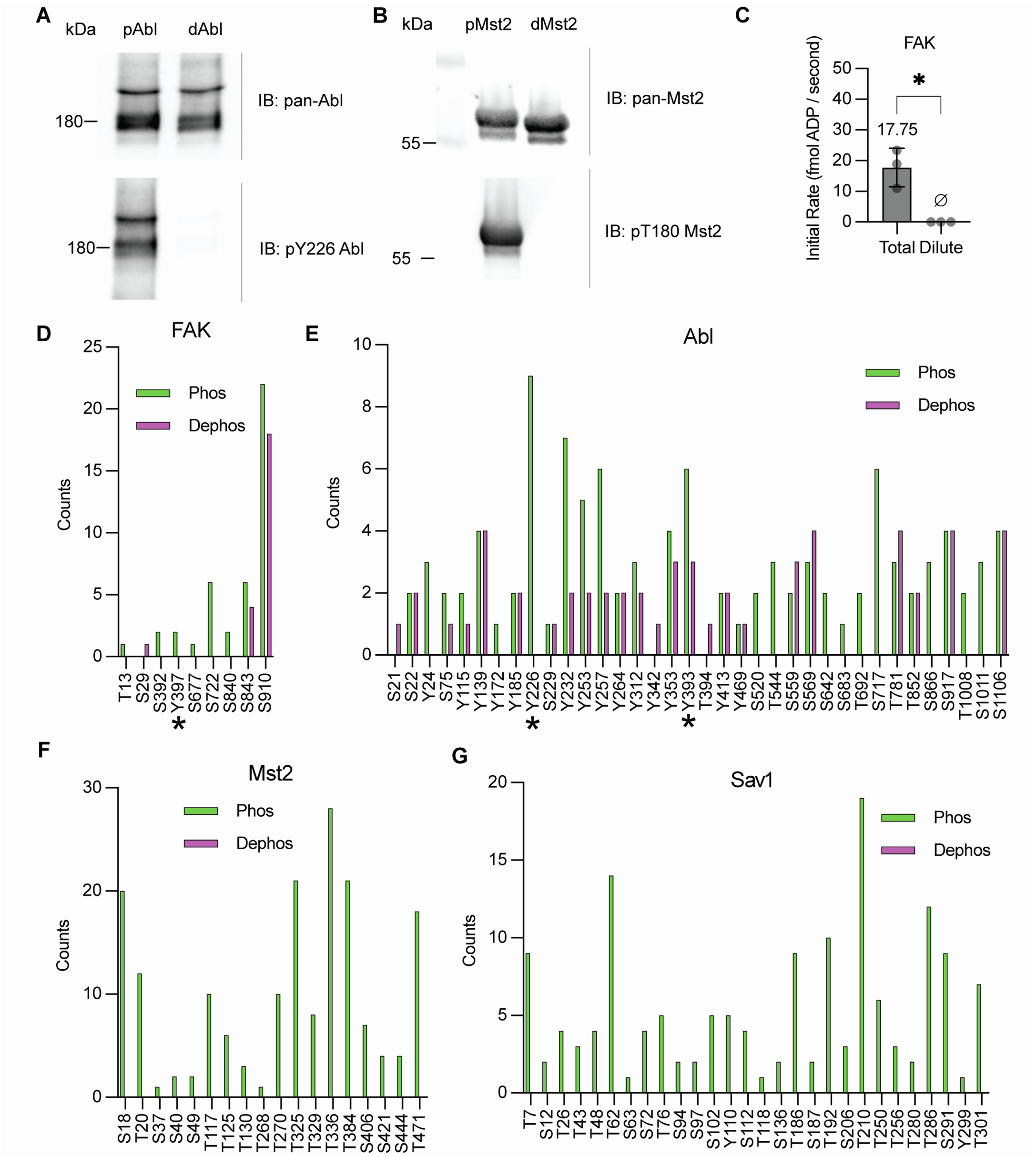

Fig. S10. FAK, Abl and Mst2 undergo autophosphorylation on multiple sites in condensates, related to Figure 3. (**A-B**) Representative western blots of Abl (A) and Mst2 (B) before and after autophosphorylation reactions. (**C**) Initial rates of FAK phosphorylation assays. Assays were performed in 25 mM HEPES pH 7.5, 50 mM NaCl, 1% glycerol (v/v), 1 mM DTT, 0.5 mM $MgCl_2$ and initiated by addition of 1 μM ATP. Error bars are standard deviation. N=3 replicates. Number above error bars is the mean. ø symbol denotes activity was undetectable. Significance was tested with unpaired t-test with Welch's correction (* p<0.0332, ** p<0.0021, *** p<0.0002, **** p<0.0001). (**D-G**) Phospho-mass spec results of before (dephos) and after (phos) phosphorylation reactions performed on total phase separated kinase solutions. Asterisks denotes canonical autophosphorylation sites where detected.

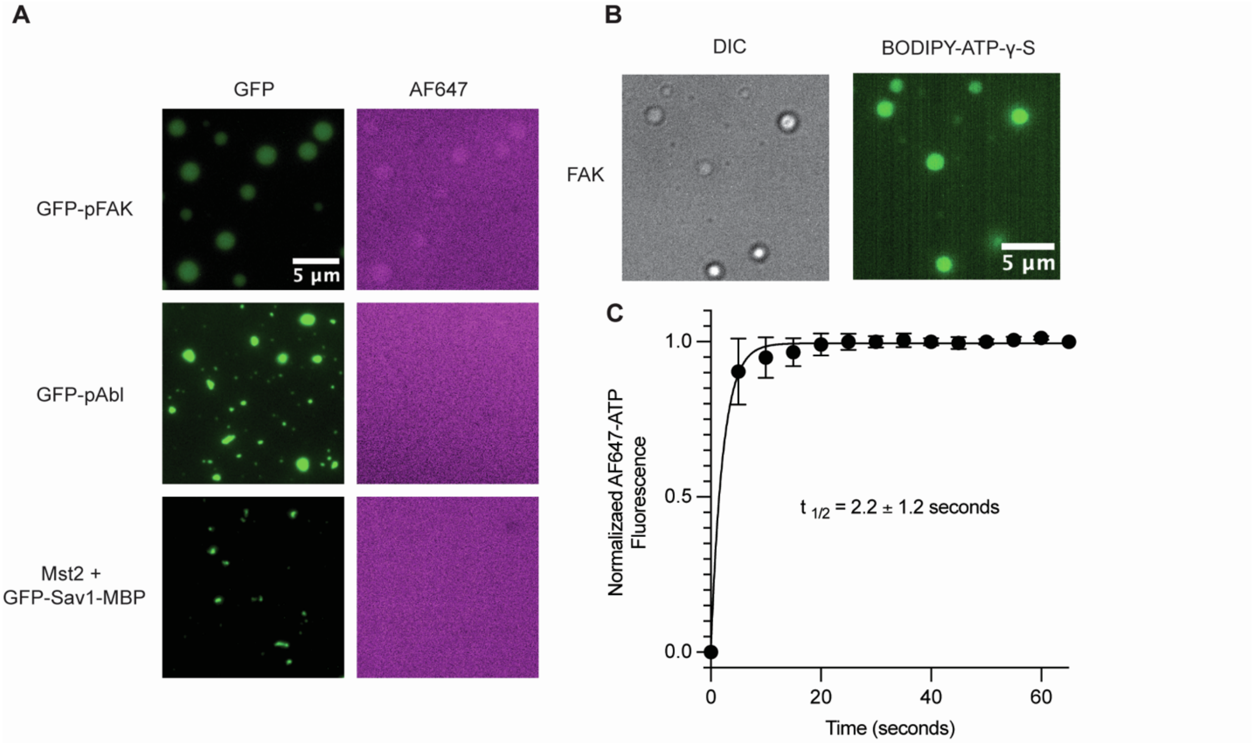

Fig. S11. Kinase condensates enrich ATP independent of dye moiety, related to Figure 4. (**A**) Microscopy images of 2.5 µM AF647 dye incubated with 1 µM mEGFP-FAK, 1 µM mEGFP-Abl or 100 nM mEGFP-Sav1-MBP + 100 nM Mst2. Final buffer conditions match those in Fig. 3A (Abl and Sav1+Mst2) and Fig. 1A (FAK). (**B**) Microscopy images of 1 µM FAK. Final buffer conditions match those in (A) except supplemented with 2.5 µM BODIPY-ATP-γ-S. (**C**) Timecourse of AF647-ATP fluorescence intensity in mEGFP-FAK condensates after addition of 2.5 µM AF647-ATP. Buffer matches that in Fig. 1B. N=7 droplets across 2 technical replicates. Error and error bars represent standard deviation. All scale bars are 5 microns.

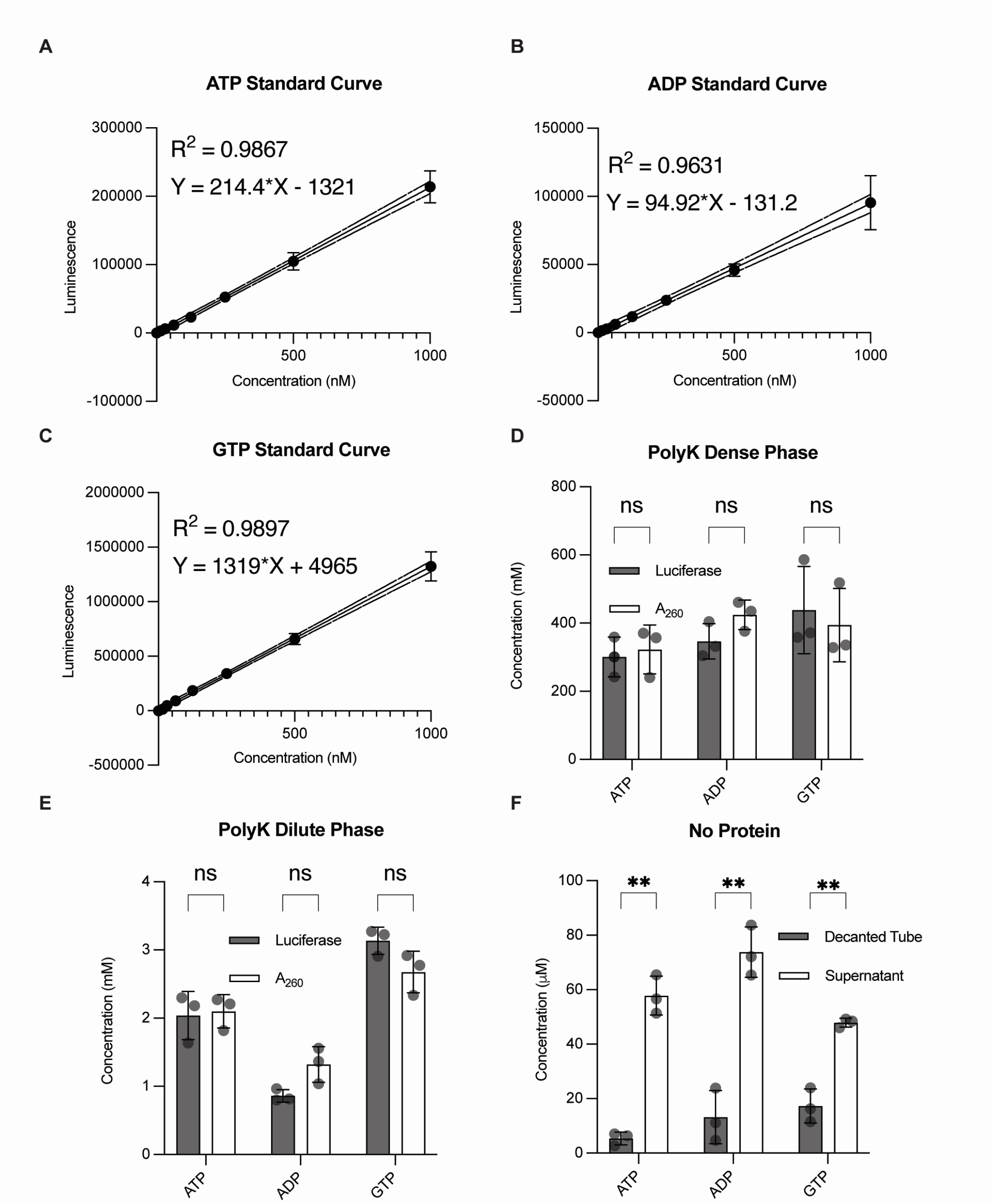

Fig. S12. Condensate sedimentation-luciferase assays accurately measure condensate nucleotide concentrations, related to Figure 4. (**A-C**) Standard curve of ATP (A), ADP (B), and GTP (C) standards in Nucleotide-Glo Assay Standard Buffer (25 mM HEPES pH 7.5, 200mM NaCl, 10 mM MgCl2, 60 mM guanidine HCl). For (A), (B), and (C) dotted lines represent 95% confidence intervals of linear regression. (**D-E**) Comparison of dense phase (D) and dilute phase (E) nucleotide concentrations measured from A260 spectrophotometry or sedimentation-luciferase assays of poly-L-Lysine condensates. Condensates were formed from ~100 μM poly-L-Lysine and 5 mM nucleotide in 10 mM immidazole pH 7.0 buffer. (**F**) Comparison of sedimentation-luciferase nucleotide measurements in solutions containing no protein. Samples taken from the supernatant and from resuspended decanted tube (capturing any nucleotides adsorbing to the tube). For all plots error bars represent standard deviation and statistical comparisons are unpaired t-tests with Welch correction (* $p<0.0332$, ** $p<0.0021$, *** $p<0.0002$, **** $p<0.0001$).

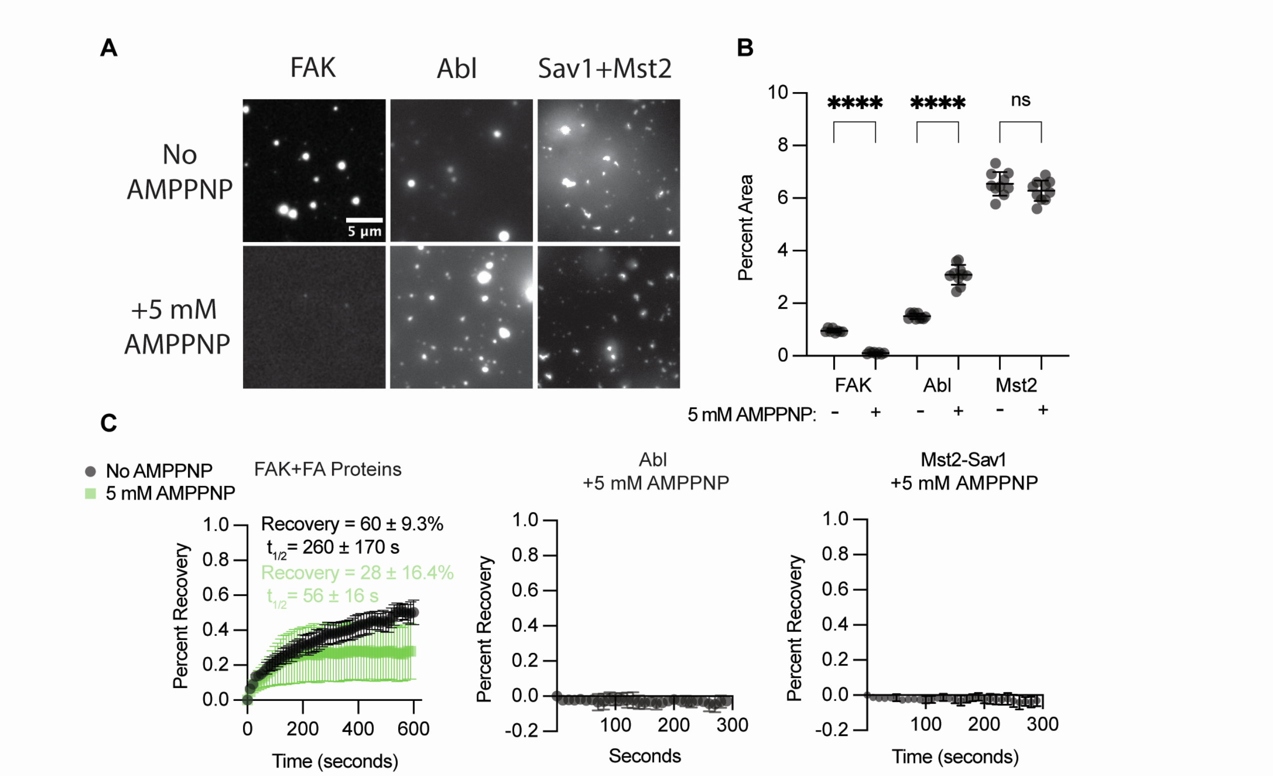

Fig. S13. Physiological ATP concentrations do not inhibit Abl or Mst2 condensate formation and does not increase FRAP recovery of kinase condensates, related to Figure 4. (**A**) Images of kinases with or without 5 mM AMPPNP. Buffers match those in Fig. 3B (Abl and Sav1+Mst2; 1000 nM) and Fig.1B (WT-FAK Low Salt). Scale bar is 5 microns. (**B**) Percent area occupied by condensates for conditions in (A). Significance was tested with unpaired t-tests with Welch's correction (* p <0.0332, ** p<0.0021, *** p<0.0002, **** p<0.0001)(N>8 images for each condition)(**C**) FRAP analysis of kinase condensates with (black) or without (green) 5 mM AMPPNP. For FAK, the FA proteins Paxillin, Nck, N-WASP, and p130Cas where added at 1 μM. All conditions used buffers identical to those used in (A). For all graphs, error bars represent standard deviation. Error bars not shown are smaller than symbol.

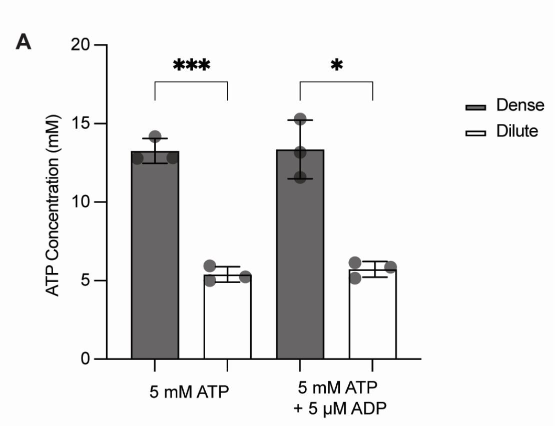

Fig. S14. Reconstituted focal adhesion condensates enrich ATP at physiological ATP and ADP concentrations, related to Figure 4. (**A**) Dense and dilute phase ATP concentration measurements of reconstituted focal adhesion condensates. FAK, paxillin, Nck, N-WASP and p130Cas are all 1 µM final concentration. Buffer: 25 mM HEPES pH 7.5, 50 mM NaCl, 1% glycerol (v/v), 1 mM DTT, 5 mM ATP with or without 5 µM ADP. N=3 replicates. Error bars denote standard deviation and statistical tests are unpaired T-tests with Welch correction (* p<0.0332, ** p<0.0021, *** p<0.0002, **** p<0.0001).

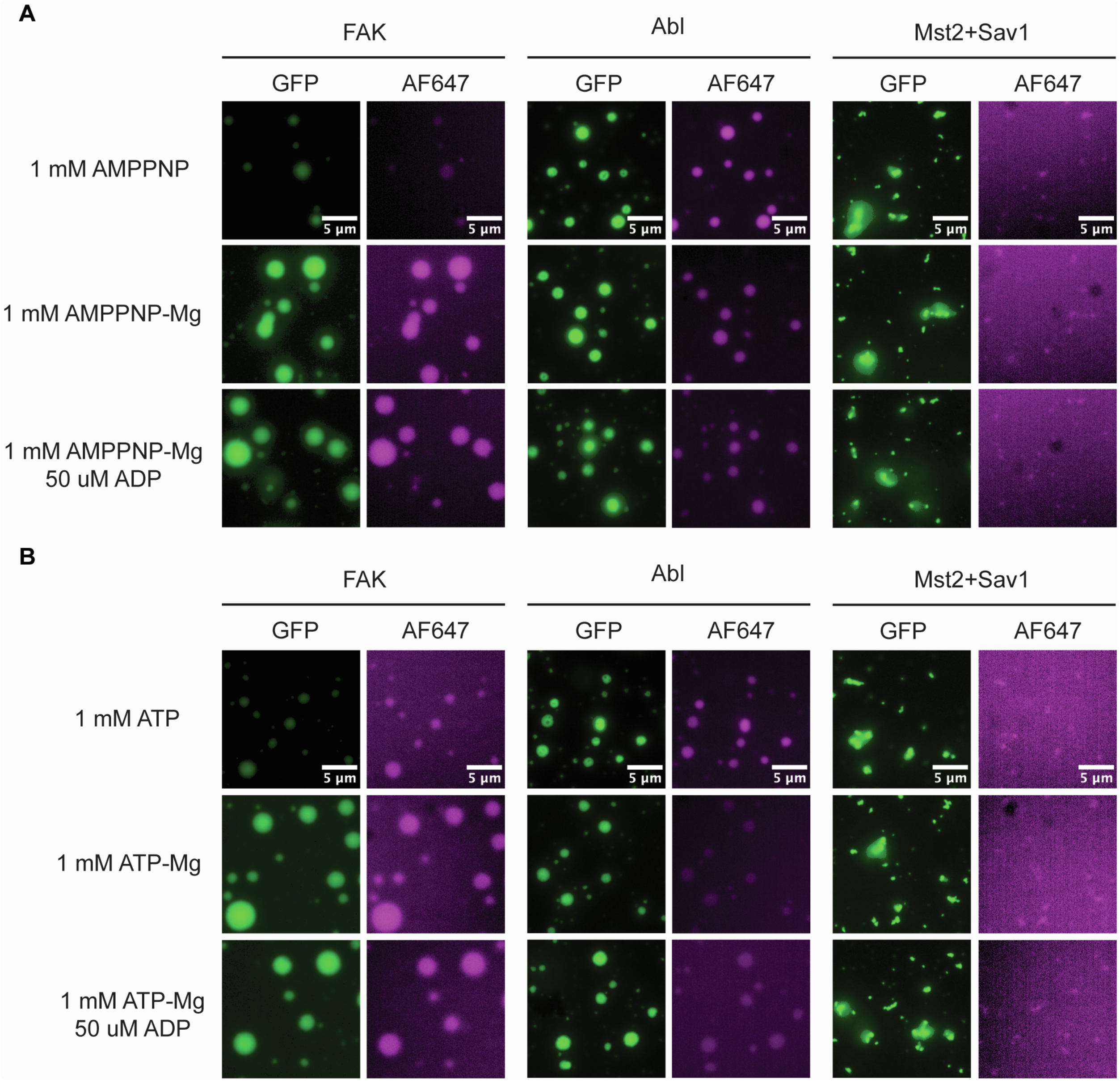

Fig. S15. ATP enrichment in kinase condensates is not disrupted by the presence of physiological Mg^{2+} or ADP concentrations, related to Figure 4. (A) Representative fluorescent microscopy images of focal adhesion, mEGFP-Abl and Mst2+mEGFP-Sav1 condensates with 1mM AMPPNP with or without 1 mM $MgCl_2$ and 50 μM ADP. Final protein concentration is 1 μM for all conditions. Focal adhesion condensates were made from mEGFP-FAK, paxillin, Nck, N-WASP and p130Cas. Buffer: 25 mM HEPES pH 7.5, 100 mM NaCl, 10 μM AF647 ATP, 1% glycerol (v/v), 1 mM DTT supplemented with 5 or 10% PEG8000 for Abl and Mst2+Sav1, respectively. (**B**) Identical conditions in (A) except with ATP instead of AMPPNP. All scale bars are 5 microns.

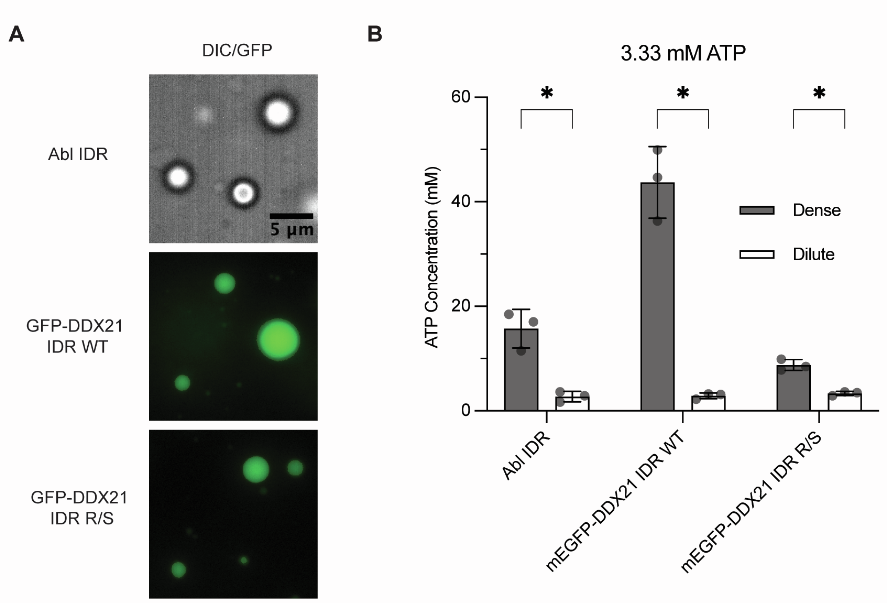

Fig. S16. Abl and DDX21 IDR condensates enrich ATP at physiological ATP concentrations, related to Figure 5. (**A**) DIC (greyscale) or fluorescence (green) images of IDR condensates. Buffer conditions are 20 µM protein, 25 mM HEPES pH 7.5, 100 mM NaCl, 20% PEG8000, 1 mM DTT and 3.33 mM ATP. Scale bar is 5 microns. (**B**) Dense and dilute phase ATP concentration measurements from sedimentation-luciferase assays. Buffer conditions are identical to those in (A). N=3 replicates. Error bars denote standard deviation. Statistical tests are unpaired T-tests with Welch correction (* $p<0.0332$, ** $p<0.0021$, *** $p<0.0002$, **** $p<0.0001$).

Methods S1. Calculation of volume-normalized fold rate enhancement of the dense phase

The percent of the total volume occupied by the dense phase (ie. droplet volume fraction; DVF) was calculated from the data in Fig. 1H and Fig. 1I using the equation $DVF = \frac{[Total] - [Dilute]}{[Dense]-[Dilute]}$ (see derivation below). Since the experiments in Fig. 1G used identical conditions and total FAK concentrations, this DVF was used to determine the volume-normalized fold rate enhancement of the dense phase ($VNFRE_{dense}$) using the equation $VNFRE_{dense} = \frac{(\frac{Dense\ rate}{DVF})}{(\frac{Dilute\ rate}{(1-DVF)})}$. The $VNFRE_{dense}$ was calculated for each of the 3 replicates in Fig. 1G generating N=3 values plotted in Fig. 1J. The dilute rate was measured directly, while the dense rate was determined by subtracting the dilute rate from the total rate.

Derivation of $DVF = \frac{[Total] - [Dilute]}{[Dense]-[Dilute]}$

V_{Den} = volume of dense phase
V_{Dil} = volume of dilute phase
V_{Tot} = total solution volume
$[Dense]$ = dense phase concentration
$[Dilute]$ = dilute phase concentration
$[Total]$ = total solution concentration
DVF = droplet volume fraction; percent of total solution occupied by dense phase

By definition:

$Eq. 1$ $$DVF = \frac{V_{Den}}{V_{Tot}}$$

$Eq. 2$ $$V_{Tot} = V_{Den} + V_{Dil}$$

Dividing each side of *Eq. 2* by V_{Tot} yields:

$$1 = DVF + \frac{V_{Dil}}{V_{Tot}}$$

Subtracting DVF from each side yields:

$Eq. 3$ $$1 - DVF = \frac{V_{Dil}}{V_{Tot}}$$

By conservation of mass:

$$(V_{Den})[Dense] + (V_{Dil})[Dilute] = (V_{Tot})[Total]$$

Dividing each side by (V_{Tot}) and substituting in *Eq. 1* and *Eq. 3* yields:

$$DVF[Dense] + (1 - DVF)[Dilute] = [Total]$$

Distributing $[Dilute]$ yields:

$$DVF[Dense] + [Dilute] - (DVF)[Dilute] = [Total]$$

Subtracting $[Dilute]$ from both sides and factoring out DVF on the left side yields

$$DVF([Dense] - [Dilute]) = [Total] - [Dilute]$$

Finally, dividing both sides by $([Dense] - [Dilute])$ yields

$$DVF = \frac{[Total] - [Dilute]}{[Dense] - [Dilute]}$$

Using our measured values of $[Dilute]$ = 0.0452 μM and $[Dense]$ = 106 μM, and knowing our total concentration of FAK added in each experiment (1 μM) the DVF is calculated to be 0.9%. We believe this to be a conservative upper bound of the DVF under these conditions since our measured $[Dense]$ value is likely smaller than the actual value. This is because under these conditions FAK forms relatively small (<5 μm) droplets which likely yield arbitrarily smaller mean intensity values due to the point spread function. Therefore, we also believe our calculated $VNFRE_{dense}$ values are conservative lower bound estimates, since a lower DVF values would yield a larger $VNFRE_{dense}$ calculation.
